# Cognitive impairment and danger to humanity, or personal assistant and learning tool? Exploring attitudes towards ChatGPT and their predictors

**DOI:** 10.1371/journal.pone.0341693

**Published:** 2026-10-08

**Authors:** Ana Jovančević, Izabel Cvetković, Jasper Van Assche, Mladen Popović, Eva Bittner

**Affiliations:** 1 Lero, the Research Ireland Centre for Software, University of Limerick, Ireland; 2 Department of Psychology, University of Limerick, Ireland; 3 Department of Psychology, University of Niš, Serbia; 4 Department of Informatics, University of Hamburg, Germany; 5 Center for Social and Cultural Psychology (CESCUP), Université Libre de Bruxelles, Belgium; 6 Optentia Research Unit, North-West University, Vanderbijlpark, South Africa; 7 Department of Anglistics, University of Niš, Serbia; Dental Hypothesis, IRAN, ISLAMIC REPUBLIC OF

## Abstract

This study examines the factors shaping attitudes towards ChatGPT inclusive of personality traits, ideological orientations, AI literacy, gender, and cultural context. In Study 1 (*N*_total_ = 500; *N*_valid_ = 472 Serbia), positive perceptions of ChatGPT as a learning tool and personal assistant were linked to higher AI literacy and supportive views of AI, whereas negative AI-related attitudes predicted concerns about ChatGPT’s cognitive impairment and existential threats. Gender played a crucial role: among men, higher levels of right-wing authoritarianism (RWA) and social dominance orientation (SDO) predicted more negative attitudes, while among women, RWA, SDO, and Openness were associated with more positive views. Study 2 compared German (as a representative of one of the most developed countries, especially in AI development; *N*_total_ = 115; *N*_valid_ = 112) and Serbian (as an example of a less AI-developed country; *N*_total_ = 119; *N*_valid_ = 117) samples, revealing significant differences (e.g., Germans scored higher in AI literacy, while Serbian participants scored higher in Conscientiousness and Extraversion. We further found different and unique effects of the examined predictors on participants’ AI- and ChatGPT-related attitudes across both samples, possibly driven by the above-mentioned base differences. These findings underscore a complex interplay of individual and cultural factors that drive divergent interpretations of AI’s societal role, highlighting the need to consider context when assessing public acceptance of emerging technologies.

## Introduction

The field of artificial intelligence emerged in the 1950s and its continual development since then [1, 2] produced ChatGPT as one of its outcomes. ChatGPT is an unsupervised language model where an unsupervised learning paradigm was used during pre-training, and no fine-tuning and RLHF stages were applied afterwards. It can provide input on various topics, and in various languages [3]. Attitudes towards this tool as seen on social networks vary a lot, thus, in this study, we aim to examine these attitudes as well as their potential predictors. ChatGPT is an important tool to study due to its vast usage, which boomed very soon after its release, opening up completely new avenues in work, research, education, and medicine. Due to the high number of users and ways of usage, it is crucial to understand and learn more about the characteristics of individuals prone to the misuse this (relatively) novel tool. Hence, the main aim of this article is to examine potential predictors of attitudes towards ChatGPT and investigate their role in two different cultures: Serbia and Germany.

The formation of our model was influenced by articles showing that people have mixed opinions about AI [4], with said opinions explainable in part by individual differences like personality traits [5], authoritarianism [6], social dominance orientation (Pauketat & Anthis, 2022), and need for cognitive closure [7]. This can be attributed to the fact that few studies are aimed at ChatGPT in particular. Depending on the level of these constructs present in the individual, attitudes towards AI vary. In addition to psychological dispositions, some of the crucial differences come from: 1) AI literacy—one of the most important predictors of positive attitudes towards AI [8]; 2) gender [9,10]—with men having more positive attitudes; and 3) culture [11]. These outlined variables align with the dual-process theory of attitudes [12,13], since we have explicit attitudes (or judgments) in AI literacy (deliberate process) and implicit attitudes present in the form of psychological characteristics, demographics, and culture.

Research shows that higher scores on different personality traits could relate to AI literacy in different ways [14]. Stenner [15] argues that authoritarian individuals have a strong need for order, predictability, and clear social hierarchies. They resist ambiguity and complexity, as these threaten their sense of stability and control. As a result, they may be more sceptical of AI in general and ChatGPT in particular. Individuals with high need for cognitive closure [16] and social dominance orientation [17] may also differ in their outlook towards AI in comparison to those with low scores on these variables. The details of all these connections will be discussed in detail in literature review part.

Finally, some factors may moderate this relationship. In this paper, we outline three moderating factors: gender, AI literacy, and culture.

Gender is hypothesised as moderator due to gender differences in: 1) personality traits [18]; 2) authoritarianism (RWA; [19])—with some studies showing that women endorse authoritarian values more than men; 3) social dominance orientation (SDO; [20])—with women having lower SDO in general; 4) need for cognitive closure [21]—with women having a higher dispositional tendency of structure, preference for secure knowledge and discomfort with uncertainties; and 5) attitudes towards AI [9,10]—with man having more positive attitudes.

Another possible moderating factor is AI literacy, a crucial predictor of positive AI attitudes [8], due to differences in: 1) personality, with different correlations between the various personality traits and computer literacy [22], with Openness, Conscientiousness, Extraversion, and Agreeableness having a positive correlation with computer literacy, and Neuroticism a negative one; 2) gender disparities in AI usage and differences in self-assessed knowledge [23]; and 3) culture, with German students having higher AI literacy than UK and US students [24].

On a final note, cultural differences are pervasive and encompass differences in: 1) personality (e.g., [25]); 2) RWA (e.g., [26]); 3) SDO (e.g., [27]); 4) NFC (e.g., [28]); and 4) attitudes towards AI [11]. A comparison between Serbia and Germany is relevant due to the relative collectivist versus individualist nature of these respective countries, and particularly due to the spatial gap existing between these two countries in terms of interest in AI in general [29]. Going further, Germany has a very developed AI community, being one of the first countries with a developed AI strategy [30], while also using AI in education more and more every day [31]. In contrast, Serbian development in terms of AI is lacking in comparison to Germany. Also, regarding other indices, Serbia’s can considered as similar to other less developed countries (as per, for example, corruption index, freedom of the press, brain drain, and more; [32–34], while Germany can be seen as a representative of western/more developed cultures. It would therefore be of great relevance to compare countries with such disparities in terms of culture as well as AI adoption.

Finally, we assume that attitudes towards AI in general will not be completely identical to the attitudes towards ChatGPT as a particular tool, as AI has numerous applications, many having very different affordances in comparison to a conversational AI tool such as ChatGPT.

Current studies cover a wide range of possible predictors and moderators in predicting attitudes towards AI and technology in general but are lacking in systematic examination of all of these possible variables together—that is, examining their joint effects and interactions. Further, previous studies did not examine cultural differences of the full model of possible predictors—another gap this study aims to fill. Finally, research regarding specific tools like ChatGPT is generally lacking, which constitutes the third gap we aim to address in this study. Thus, we propose a model in which personality traits, RWA, SDO, and NFC are predictors of attitudes towards AI in general and ChatGPT in particular, while gender, AI literacy and culture are moderating factors.

## Literature review

### AI: A short overview

Since the early developments of AI in the 1950s, AI technologies have proliferated in multiple fields, including education, robotics, and natural language processing [1,2]. In recent years, conversational AI has garnered particular attention due to its interactive capabilities and broad accessibility (Collins et al., 2021). Among the latest tools in this domain is ChatGPT (Chat Generative Pre-Trained Transformer), a large-scale language model released by OpenAI, which amassed over 100 million users within months of its debut, with the first million surpassed in just five days from its release [3]. Its striking uptake is reflected not only in general usage statistics but also in its emerging applications for interactive learning and educational support [35].

ChatGPT is part of OpenAI’s GPT-n series, a family of large language models that have successively increased in scale and capability. GPT-1 was trained on approximately 7,000 novels, chosen for their extended narrative structure [36]. GPT-2 further expanded its training to eight million web pages, while GPT-3 dramatically scaled up to 499 billion tokens (equalling around 570 GBs of data compared to GPT-1s 4.5 and GPT-2s 40 GBs; [37]. By incorporating even larger datasets and refined architectures—some aspects of which OpenAI has not publicly disclosed—GPT-3.5 and GPT-4 underscore the continual growth of these foundational models [38]. ChatGPT leverages these successive enhancements, employing transformer-based architecture and reinforcement learning from human feedback to produce coherent and contextually adaptive responses [39]. Its defining strength lies in how it melds generative text capabilities with a conversational interface, enabling both casual and specialized users to interact in ways previously limited to human dialogue [40].

This research focuses on ChatGPT because it combines two elements that make it especially salient for studying attitudes towards AI. First, it offers novel interactive features: users can engage in near-human-like conversations on various topics, giving ChatGPT the potential to transform how people learn, collaborate, and problem-solve. Second, it sits at the forefront of public discourse on AI, illustrating both the possibilities (e.g., improved literacy, tailored feedback) and the concerns (e.g., data privacy, credibility of generated information) that accompany large-scale AI adoption. Understanding how individuals perceive ChatGPT is thus an important step in mapping broader attitudes towards AI-driven tools—particularly those that expand educational opportunities and shape public trust in automated decision-support systems.

Because ChatGPT stands at the intersection of large-scale language modelling, practical real-world adoption, and public debate, it warrants focused investigation into how attitudes towards AI manifest when users interact with a system that closely imitates human conversational patterns. The following chapters will therefore examine how these attitudes may converge with—or diverge from—general views of AI, shedding light on users’ acceptance, concerns, and overall relationship with a high-profile generative language model.

### Attitudes towards AI

Public opinion on AI has long been polarized, with positive attitudes focusing on potential benefits, such as enhancing efficiency and supporting human professionals [41], alongside neutral or negative views tied to data privacy, distrust of corporate motives, and concerns about fairness or job displacement [4]. These mixed opinions often reflect the tension between perceived advantages (e.g., convenience, problem-solving capacity) and perceived risks (e.g., ethical dilemmas, immature technology; [42]).

Although these insights are valuable for understanding how people generally feel about AI, fewer studies have examined attitudes towards specific AI tools like ChatGPT. Its conversational interface and large-scale language modelling mean that user responses may differ from general attitudes towards AI. Individuals who view AI cautiously in other settings might still appreciate ChatGPT’s real-time assistance; conversely, those who generally trust AI might question ChatGPT’s outputs if they perceive them as inaccurate, biased, or lacking credible safeguards. This underscores the importance of exploring whether and how general attitudes towards AI carry over to ChatGPT, given the system’s distinct capabilities and wide adoption.

By focusing on this specific context, we aim to uncover how individual differences, ranging from personality traits to ideological views, relate to ChatGPT. Establishing these links will help to clarify the ways in which broader scepticism or enthusiasm for AI can manifest in the more personal, interactive domain of a text-based tool that users can engage with daily.

### Personality traits

Personality traits can determine the nature of Human-AI interaction, starting from attitudes towards AI [5], AI-assisted hiring practices [43], general AI acceptance by HR workers [44], and business workers [45]. For example, a recent study found that personality can predict attitudes towards AI [5]. Introverts are more positively orientated towards AI and more forgiving for its negative aspects, potentially due to reduced social contact [46]. Conscientiousness showed a positive correlation with negative attitudes towards AI [5], while high openness to experience is connected to a higher trust level [47].

Unlike the association between AI-related attitudes and personality traits, the relationship between personality traits and AI adoption seems more straightforward. Research consistently shows that all personality traits, except for neuroticism, affect the easiness of AI adoption [43–45].

Emerging studies also explore the HEXACO personality framework in this context (which we used in this study as well). This framework consists of six dimensions: Honesty-Humility, Emotionality (akin to Neuroticism in the Big Five Framework), Extraversion, Agreeableness, Conscientiousness, and Openness to Experience [48]. Studies have already examined the relationship between the HEXACO framework and attitudes towards AI. For example, one study found that support for strict regulation of AI is positively related to Agreeableness, Neuroticism, and Honesty–Humility [49].

While Openness and Conscientiousness broadly shape attitudes towards AI (e.g., trust in autonomous systems or robots), ChatGPT—as a conversational agent—introduces unique dynamics. General AI scepticism among conscientious individuals often stems from abstract ethical concerns (e.g., job displacement, bias), but ChatGPT’s interactive and linguistic nature amplifies specific anxieties, such as distrust in its factual accuracy (“hallucinations”) or discomfort with its mimicry of human communication. Similarly, while introverts may favour AI broadly for reducing social demands (e.g., automated services), ChatGPT’s role as a socially adjacent tool—capable of replacing human dialogue—may deepen this preference, as introverts avoid the emotional labour of interpersonal exchanges. Conversely, traits like Agreeableness, which minimally predict general AI attitudes, could become salient with ChatGPT if users perceive its responses as lacking empathy or ethical nuance. Thus, ChatGPT’s conversational fluency and ambiguity in intent (e.g., “Is it a tool or a pseudo-social agent?”) create distinct trait-driven tensions absent in non-linguistic AI-applications, where functionality dominates over social or ethical rapport. As our study focuses on a specific tool, this narrower focus may reveal different patterns compared to broader research on AI-adoption or general attitudes towards AI. Positive attitudes towards a particular tool (which people can choose to use or not) might more directly correlate with its actual usage, as opposed to attitudes towards AI as a whole (which may portrait ideal AI and imagined usage).

## Ideology

### Authoritarianism

RWA reflects a preference for social order, tradition, and deference to authority (Altemeyer, 1981). A study conducted by Asbrock and colleagues [6] revealed that authoritarianism is connected to attitudes towards AI. More precisely, authoritarianism showed a small positive total effect on attitudes towards the use of AI in autonomous vehicles and a strong positive relationship with attitudes towards the use of AI in surveillance [6]. The same study suggests that authoritarianism shows an ambivalent relationship with AI attitudes, meaning that individuals high in this trait have both positive and negative attitudes [6]. Some research further suggests that people with more conservative attitudes are more sceptical of AI in comparison to liberals [50]. There is currently a lack of studies examining the relationship between authoritarianism and conversational AI like ChatGPT, and we can assume a different relationship than with other AI technologies due to double standards these individuals have in general [51]. Since ChatGPT (at least allegedly) gives out information freely, not necessarily supporting authoritarian views, it may not be viewed in a positive manner as other AI technologies. Acceptance/rejection of ChatGPT by these individuals could relate to their general rejection of science and new scientific discoveries [52], which we aim to explore in this study.

### Social dominance orientation

We will also investigate the possible role of social dominance orientation (SDO). SDO relates to preference for inequality between groups and preference for hierarchical social structure [53]. As it stands, SDO could potentially have divergent implications for perceptions of conversational AI as ChatGPT based on different facets and specific attitudes towards the tool. For example, individuals high in SDO could show lower preference for ChatGPT implicating introducing lower inequalities and disturbing hierarchical disbalance in society. Since SDO is correlated with the moral prioritization of humans over animals [54] we aim to understand its relationship to attitudes concerning a specific form of AI – ChatGPT, in the context of prioritisation of humans over AI. Further, SDO is associated with more selfish behaviours [55] which could possibly entail using ChatGPT for one’s own gain. The specific direction of the relationship is thus somewhat unclear, it seems however to be leaning towards a negative one. We could hypothesise that AI tools can help bridge the inequalities in society and thus could be rejected—the same way individuals with high SDO may reject science and new scientific discoveries [52], supported by research showing that conservative individuals may hold negative attitudes towards technological developments [56].

### Cognition

Another important variable is the need for cognitive closure (NFC). This is a motivated tendency that includes various aspects, like desire for predictability, preference for order and structure, discomfort with ambiguity, decisiveness, and close-mindedness [16]. Previous studies have shown that a high need for closure is associated with more negative attitudes towards AI [7]. Specifically, they have found that discomfort concerning ambiguity was related to negative attitudes towards robots [7]. Therefore, the need for closure should be incorporated into research regarding attitudes towards AI.

### Moderating factors

Several factors, such as AI literacy, gender, and culture, may moderate the relationships described above. Since these factors are relatively objective and are largely independent of psychological influences, we will consider them as moderators, following the approach used in the Unified Theory of Acceptance and Use of Technology [57]. In addition, as we stated in the introduction, these factors not only differentiate attitudes towards AI, but also between psychological predictors in our model.

### AI literacy

Previous studies show that knowledge concerning AI is associated with the intention to actually use AI technology [58]. Individuals with low knowledge of AI have mostly negative attitudes about it, with most of the concerns being who has access to the AI’s data and when [8]. One study confirms that AI literacy is strongly related to digital literacy, attitudes towards robots, and the daily use of AI [59]. Another study shows that perceptions of AI differ based on the participant’s gender, age, knowledge of AI, and experience with AI [10].

AI literacy is thus hypothesised to be a moderator in the relation between individual differences and attitudes towards ChatGPT. For example, individuals high in NFC crave predictability and dislike ambiguity [60]. When encountering AI chatbots, those with low AI literacy may perceive the technology as a “black box,” amplifying anxiety about its unpredictability. High AI literacy, however, equips individuals with knowledge about how AI (including chatbots) operates, thus reducing unpredictability [61]. For individuals high in RWA, AI chatbots may initially seem threatening if perceived as destabilizing societal norms (e.g., replacing human roles or spreading “woke” biases). Drawing on System Justification Theory [62], literacy may highlight AI’s utility in upholding authority structures and therefore strengthen the positive association between RWA and AI-attitudes. As for personality traits, AI literacy may serve as a buffer for some, or have an amplificatory effect for others. For example, individuals with high openness to experience typically embrace novelty [14]. High AI literacy may deepen their engagement by highlighting creative applications (e.g., coding assistance, brainstorming), amplifying positive attitudes. Similarly, conscientious individuals prioritize reliability [14]. High AI literacy clarifies chatbots’ error rates and limitations (e.g., “hallucinations”), enabling informed trust. Without literacy, conscientious users might overestimate risks, leading to avoidance. Individuals with high neuroticism experiencing anxiety about AI (e.g., job displacement, privacy) may be tempered by literacy that contextualizes risks (e.g., explaining safeguards like anonymization). This aligns with Cognitive Appraisal Theory [63], where knowledge reframes threat perceptions.

Literature on exact nature of moderating effect of AI literacy on variables in our study is lacking; hence we can’t hypothesize the exact direction of the moderating effects precisely, but we can assume that moderation will be significant, based on the above-mentioned literature. Hence exploring the exact nature of moderation is one of the novelties in our study.

### Gender

A study conducted on a student sample showed that men report higher readiness to use AI, confidence in AI’s abilities, and higher perceived relevance, in comparison to women [9]. In a study conducted on medical students, men agreed at a higher rate that AI could potentially automatically detect pathologies in imaging examination [64]. Women were shown to be less likely to support AI in some cases, for example, planning for a natural disaster, but not in all cases [65]. From previously mentioned studies, we can see that women are less likely to support AI in comparison to men. Similarly, research shows that gender may moderate relationships between psychological factors and intentions to use AI in situations like self-regulated learning with a chatbot [66]. These authors found that the effects of the need for autonomy and competence are moderated by both gender and AI knowledge, with stronger effects of the two in women when AI knowledge is low. On the other hand, the effect of the need for relatedness was moderated only by gender. In addition to these differences, as outlined in the introduction, men and women differ in all core psychological variables we measure in this study, so we can hypothesise different slope effects for both.

### Cross-cultural differences

Studies consistently show that there are cross-cultural differences in attitudes concerning AI. One such study examined the opinions of participants from 48 countries concerning a hypothetical scenario in which an AI was their direct superior [11]. The results suggest that people from less developed countries exhibit fewer negative attitudes towards the use of AI in such a scenario, with people from East Asia showing mostly trusting attitudes, while participants from Europe and North America showed either worrying or neutral attitudes [11]. Another study examined the role of emotional AI in a workplace (a type of AI which can assess the affective state of a worker; [67], and the results suggest that participants from East Asia show higher levels of trust towards emotional AI in comparison to people from Western societies [67]. Some differences may stem from the labour organization in a particular culture. For example, in one study, the authors found that the fear of losing a job due to AI was lower in Japanese in comparison to Swedish participants, which may stem from the Japanese labour market not prioritizing AI over human workers (i.e., firing workers upon AI implementation) in comparison to other places such as Sweden [68]. Another study suggests that American participants show more ambivalent attitudes towards robots in comparison to Chinese participants [69]. From the previously mentioned studies, we can conclude that some cultural differences exist, but those are mainly found when Western countries are compared to East Asian countries (where technology is quite advanced). In this study, we aim to explore differences between European countries, more specifically, Serbia as a less developed European country and Germany, as a more developed European country (differences can be confirmed by a few criteria, including those available in the worldbank [https://data360.worldbank.org/en/economy/DEU] data). A comparison between Serbia and Germany is particularly compelling due to the notable spatial disparity in overall interest in artificial intelligence (AI) between the two countries [29]. Germany boasts a highly developed AI community and was among the first nations to implement a comprehensive national AI strategy [30]. The integration of AI into the German education system is also steadily increasing [31]. In contrast, Serbia lags behind in terms of AI development and adoption. Additionally, cultural distinctions between the two countries are significant. According to Hofstede [70], Serbia scores higher on uncertainty avoidance, a cultural dimension associated with a lower likelihood of adopting new technologies, possibly due to increased perceptions of threat [71]. Taking these factors into account, it is reasonable to expect that the German sample would demonstrate a greater openness to using ChatGPT.

Based on these cultural differences we expect different results in these two cultures, inclusive of differences in general attitudes, AI literacy, but also moderating effect. Since these two cultures have not been compared before in the manner done in this study, we can’t make predict directional hypotheses regarding to the moderating effect of the culture, however exploring the nature of this moderation is one of the novelties of this study.

### Hypotheses

Based on the outlined previous studies, we draw the following hypotheses:

**H1:** Attitudes towards AI positively predict attitudes towards ChatGPT.
**H2:** Attitudes towards AI and towards ChatGPT are predicted by specific personality traits. In particular:
***H2a:*** Extraversion predicts more negative attitudes towards AI and towards ChatGPT negatively.
***H2b:*** Conscientiousness predicts more negative attitudes towards AI and towards ChatGPT.
***H2c:*** Openness predicts more positive attitudes towards AI and towards ChatGPT.
***H2d:*** Honesty-Humility predicts more negative attitudes towards AI and towards ChatGPT.
***H2e:*** Emotionality predicts more negative attitudes towards AI and towards ChatGPT.
***H2f:*** Agreeableness predicts more positive attitudes towards AI and towards ChatGPT.
**H3:** Attitudes towards AI and towards ChatGPT are predicted by specific social-ideological attitudes and motivated cognition. In particular
***H3a:*** Authoritarianism positively predicts attitudes towards AI and towards ChatGPT
***H3b:*** Social dominance orientation negatively predicts attitudes towards AI and towards ChatGPT.
***H3c:*** Need for closure (NFC) negatively predicts attitudes towards AI and ChatGPT.
**H4:** AI literacy is a significant moderator in the relationships of personality traits, ideological variables (SDO, RWA), and cognition (NFC) with attitudes towards AI and ChatGPT, with AI literacy acting as positive moderator (i.e., enhancing the paths, and shifting attitudes towards more positive ones).
**H5:** Gender is a significant moderator in the relationship of personality traits, ideological variables (SDO, RWA), and cognition (NFC) with attitudes towards AI and ChatGPT, yielding different relationships between predictor and outcome variables for men versus women.
**H6:** There are significant differences in attitudes towards AI and ChatGPT between the two compared cultures. In particular, we expect Germans to be more positively oriented towards AI and ChatGPT than Serbs.

### Study outline

This study was conducted through two pilot studies and two main studies. In the pilot studies, presented in supplementary materials (S1 File; subheadings A and B), our aim was to construct a measure for attitudes towards ChatGPT. In the first main study, we examined the potential predictors of these attitudes in the Serbian sample. The second main study examined the same predictors in the context of two countries—Serbia and Germany.

The University of Hamburg approved the studies within this manuscript. The work of the Ethics Commission underlies the legal requirements of data protection of the Universität Hamburg as well as the WMA Declaration of Helsinki, the ethical guidelines of the German Informatics Society, and the ethical guidelines of the German psychological society (ethische Richtlinien der Deutschen Gesellschaft für Psychologie e.V.), each in their current version (https://www.inf.uni-hamburg.de/en/home/ethics.html). All participants were adults, and thus able to give their own consent to participate in the research. They gave informed written consent prior to participating in the research in the online form—by checking whether or not they agree with the terms and conditions of the research.

The project outline is presented in Fig 1.

**Fig 1:**
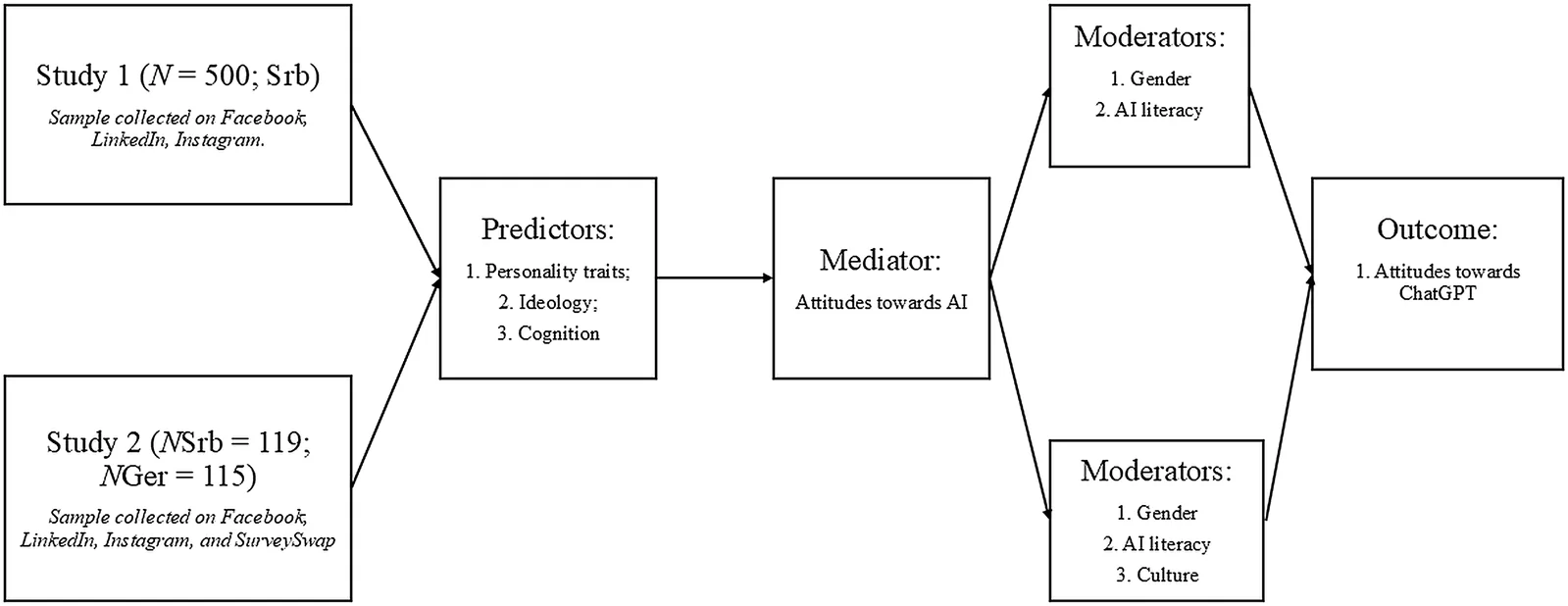
Project method outline.

## Study 1

### Method

The aim of this study was to examine the role of certain variables in the prediction of attitudes towards ChatGPT. All predictors can be divided into several groups: AI Attitudes, personality (HEXACO personality traits), Ideology (RWA, and SDO), and Cognition (NFC), with the moderating role of AI literacy and the gender of the participants. The full theoretical is presented in Fig 2.

**Fig 2:**
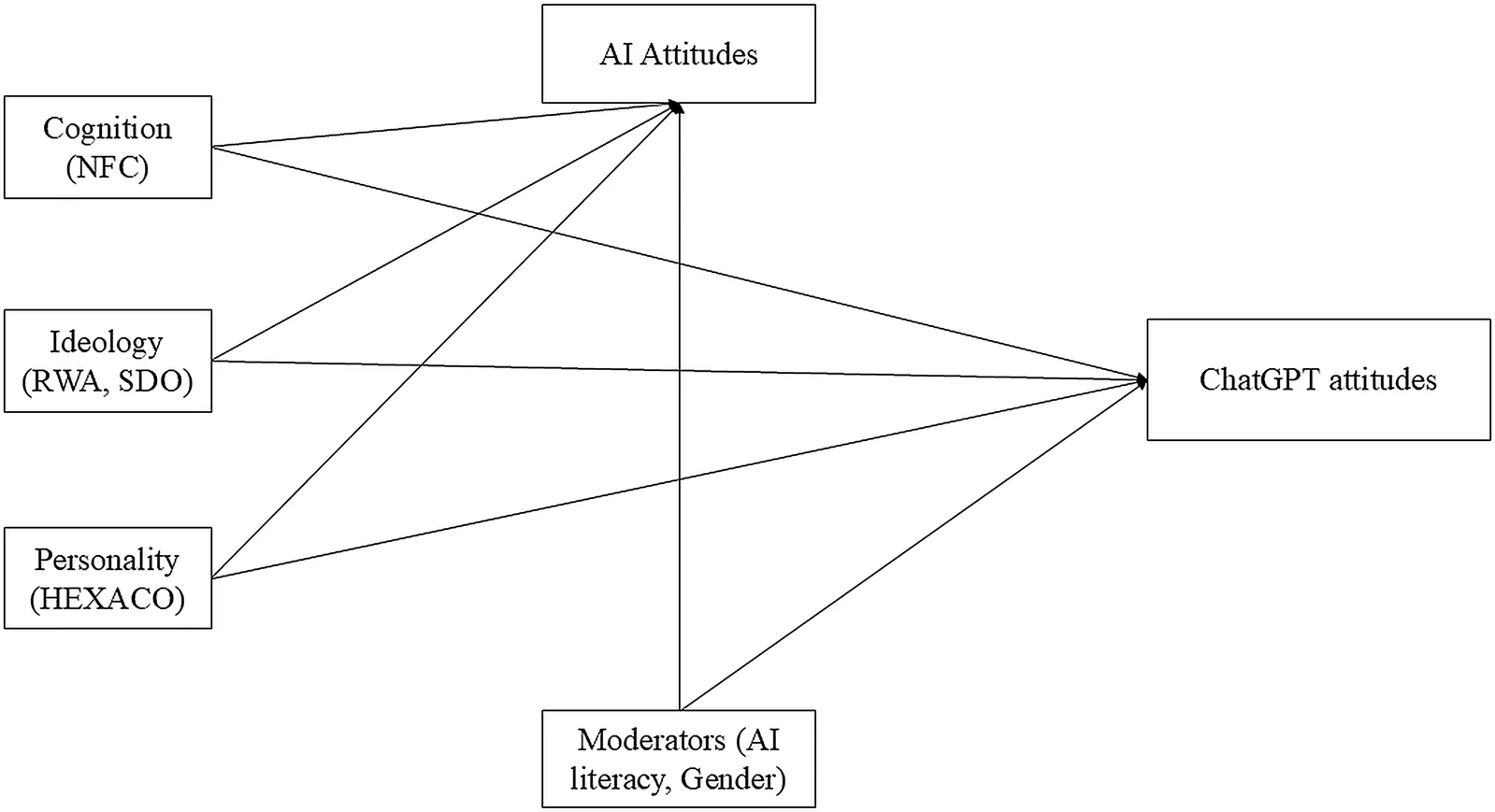
Theoretical model.

### The sample

Sample size calculation was done through GPower for *t*-tests (with power of 80%, and two tailed significance at the level of .01), suggesting sample size of 122; and through calculating a priori power for RMSEA, indicating minimum sample size of 94. Due to model complexity, we were additionally led by rules of thumb for path analysis (5:1 or 10:1 participant per parameter; Westland, 2010). As such we aimed to achieve a sample size as close as possible to the rule of 10:1. We examined 500 adults from Serbia, with an age range from 15 to 58 (*M* = 24.94, *SD* = 9.76). Twenty-seven-point four percent were men, 70.6% were women, 1.2% did not wish to disclose their gender, 0.6% were non-binary, and 0.2% were trans-men. Since gender was planned as one of our moderator variables, and given the fact that there were not enough respondents in categories non-binary and trans-men, we had to exclude them from this analysis. We also excluded those who did not wish to disclose their gender (due to their small proportion), and participants younger than 18 (*n* = 17). We also excluded one participant who didn’t agree to their data being processed. Thus, the eligibility criteria were to have enough cases for comparison, for participants to agree to their data being processes, and age (18+). This left us with 472 respondents (72% women), aged from 18 to 58 years (*M* = 25.14, *SD* = 9.76) (see Table 1 and Table 2 for further sample description). All respondents were given an informed consent form containing all information about the study. All participants agreed to participate in research. The study was assessed by a local ethics review board at the institution of one of the authors and judged not to require any further evaluation.

**Table 1:** Percentage of participants per education level in Study 1.

Category	*N*	%
Didn’t state	1	0.2
Studying towards High School Diploma	1	0.2
High School Diploma	266	53.2
College Diploma	40	8.0
Studying towards Bachelor’s Diploma	66	13.2
Bachelor’s Diploma	110	22.0
Master/PhD	16	3.2

**Table 2:** Participants’ gender in Study 1.

Gender	*N*	%
Men	137	27.4
Women	353	70.6
Does not wish to tell	6	1.2
Non-binary	3	0.6
Trans men	1	0.2

### Instruments

Before we proceed with the presentation of the scales used in this research, we want to note that the majority of the scales/dimensions analysed in this research have a small number of items. As such, they are not well suited for Cronbach’s alpha indicators, as it is expected for such short measures to show low reliability [72]. However, alternative measures can be used, such as inter-item correlations [73], which we present here.

1. *The General Attitudes towards Artificial Intelligence Scale* (GAAIS; [5]). The scale contains 20 items and 2 dimensions: positive attitudes and negative attitudes. In the original validation study, both scales had adequate internal consistency (α = .85; α = .82) respectively. The scale was checked for construct validity with confirmatory factor analysis, and for convergent and discriminant validity with related scales (e.g., Technology Readiness Index; [74]). In current study, the internal consistency was also adequate – positive (α = .90) and negative attitudes (α = 78).
2. *HEXACO* was used to operationalize personality traits (24-item version; 6 personality traits – honesty-humility, emotionality, extraversion, agreeableness, conscientiousness, and openness to experience; [75]). Internal consistency varies between α = 29 to α = 59. The average inter-item correlation varies between .17 to .27, which is considered adequately reliable [73]. Also, this inventory showed low α in the original validating study as well, but other forms of reliability were relatively high (test–retest stability, self-other agreement, and convergent correlations with full-length scales; [75]).
3. *Right-Wing-Authoritarianism (RWA)* was measured by a selection of 9 items from the Duckitt et al.’s [76] questionnaire, the same selection of items was used multiple times in previous research (e.g., [77–79]. Internal consistency reliability (α = .86) was adequate.
4. *Social dominance orientation (SDO)* was measured by three items created by [80]. The same selection of items was used in US [81] and Turkish samples [77]. Although internal consistency was low (as expected for scales with a small number of items; [72]; α = .36), but the average inter-item correlation amounted to .15, suggesting adequate reliability of the scale.
5. *Need for closure (NFC)* was measured by the selection of five items from the scale created by Roets et al. [82], with same selection of items used in another article in preparation [83]. Internal consistency reliability was lower than adequate (α = .53). The average inter-item correlation was .18, however, which suggests adequate reliability [73].
6. *AI literacy* measure [84]. This is an objective measure of AI literacy, consisting of theoretical questions. The measure has two higher-order and four-lower order dimensions. The two higher-order dimensions are Part of Human-AI interaction, and Type of Human-AI interaction. Part of Human-AI interaction further consists of Socio AI literacy and Technical AI literacy, and Type of Human-AI interaction consists of Creator/Evaluator AI literacy and User AI literacy. Discriminant, convergent, and nomological validity were adequate in the validation study. Part of Human-AI interaction is the higher-order dimension used in this study, since the original authors found this dimension to be suitable for lay users. This is an objective-knowledge type test. Out of 8, we chose 4 items most suitable for lay participants. The rationale behind the choice of the items is explained in Pilot study 2 in the supplemental materials.
7. To operationalise attitudes towards ChatGPT, we used the scale created in pilot studies 1 and 2 (available in S1 File supplementary material), with 17 items and four dimensions: ChatGPT as a personal assistant (α = .87), ChatGPT as a learning tool (α = .73), ChatGPT as a cognitive impairment risk (α = .84), and ChatGPT as a danger to humanity (α = .68). Only ChatGPT as a danger to humanity has an internal consistency reliability lower than .70. However, the average inter-item correlation for this dimension is .42, which is considered to indicate adequate reliability [73].

### Procedure

The sample was collected and administered via Google forms. Students from the University of Niš helped in the sample collection in exchange for extra course credit. Besides this source, the respondents have also been collected through social networks (Facebook, Instagram, and LinkedIn).

### Data analysis

The data were analysed using SPSS, JASP, and AMOS. SPSS and JASP were used for descriptive statistics and correlation analysis, while AMOS was used to analyse the path model.

### Results

Descriptive statistics are shown in Supplementary materials (S1 File; Table 1C). We first explored the differences between men and women in the context of positive and negative attitudes towards AI and ChatGPT through a *t*-test. The results suggest significant differences in Positive attitudes towards AI (*t*(470) = 2.177, *p* = .04, *M*difference = .184; CI 95% [.008, 360]; Cohen’s d = .223), with men having the higher score; in Negative attitudes towards AI (*t*(470) = −1.676, *p* = .048 (one sided *p* is significant), *M*difference = −.128; CI 95% [−.277, 022]; Cohen’s d = −.175), with women having the higher score; and in ChatGPT as a cognitive impairment risk attitudes (*t*(470) = −3.459, *p* < .001, *M*difference = −.39; CI 95%[−.613, −.168]; Cohen’s d = −.381), with women again having the higher score. This *t*-*t*est compared men and women on attitudes towards AI in general as well as in regard to attitudes towards ChatGPT in particular. These results show that men have more positive and less negative attitudes towards AI in general, and when it comes to ChatGPT in particular, women think ChatGPT represents a risk for cognitive impairment more than men do. The results of the *t*-*t*est thus confirm our hypothesis of gender differences.

After the *t*-test, we conducted a path analysis in AMOS to verify the model presented at the end of the theoretical introduction segment of this paper. Path analyses conducted in this research include data-informed modifications; thus, they should be considered as exploratory and future studies should aim to replicate them in a confirmatory way.

The data for the first model suggests that the model does not fit the data well: Χ² = 1291.538, *df* = 328, *p* < .01. Χ²/*df* = 3.938, GFI = .838, NFI = .587, IFI = .656, CFI = .602, RMSEA .079, SRMR = .097. The next step included adding all theoretically expected covariances (e.g., among personality traits, personality traits with their respective interaction variables, etc.). Hence, the added covariances included only theoretically expected correlations between predictor variables. The final model also excluded insignificant paths, as the number of paths in first model was high (i.e., it included all possible paths; and not all possible covariates). The final model showed an adequate fit for the data (Χ² = 476.340, *df* = 328, p < .01. Χ²/*df* = 1.452, GFI = .930, TLI = .871, IFI = .938, CFI = .930, RMSEA .031, SRMR = .073). The standardized estimates of the significant path are shown in Table 3.

**Table 3:** Estimates of the path analysis in Study 1.

Criterion variable	Direction	Predictor variable	Men		Women	
			Estimate	*p*	Estimate	*p*
Positive attitudes	<---	AI_RWA	.067	.420	−.197	<.001
Positive attitudes	<---	RWA	−.214	.018	−.039	.499
Positive attitudes	<---	SDO	.168	.056	.158	.003
Positive attitudes	<---	Openness	−.008	.927	.127	.016
Positive attitudes	<---	AI Literacy	.243	.007	.134	.013
Negative attitudes	<---	AI_SDO	−.106	.417	−.121	.043
ChatGPT as cognitive impairment risk	<---	Negative attitudes	.301	<.001	.498	<.001
ChatGPT as a personal assistant	<---	Positive attitudes	.695	<.001	.582	<.001
ChatGPT as cognitive impairment risk	<---	Positive attitudes	−.199	.006	−.030	.551
ChatGPT as a danger to humanity	<---	Positive attitudes	.027	.719	.117	.019
ChatGPT as a learning tool	<---	Positive attitudes	.717	<.001	.591	<.001
ChatGPT as a danger to humanity	<---	Negative attitudes	.521	<.001	.498	<.001
ChatGPT as a personal assistant	<---	AI_RWA	.231	<.001	−.043	.344
ChatGPT as a personal assistant	<---	AI_NFC	.041	.414	.084	.015
ChatGPT as a personal assistant	<---	AI_SDO	.031	.621	.091	.025
ChatGPT as a personal assistant	<---	Openness	.055	.362	.126	.003
ChatGPT as a personal assistant	<---	Agreeableness	−.047	.393	−.072	.043
ChatGPT as cognitive impairment risk	<---	AI_RWA	−.035	.632	.136	.004
ChatGPT as cognitive impairment risk	<---	RWA	.321	<.001	.093	.069
ChatGPT as cognitive impairment risk	<---	SDO	.221	.006	−.110	.020
ChatGPT as cognitive impairment risk	<---	Openness	.218	.003	.063	.187
ChatGPT as cognitive impairment risk	<---	AI_EX	−.010	.872	−.097	.016
ChatGPT as a danger to humanity	<---	AI_RWA	.009	.913	.111	.019
ChatGPT as a danger to humanity	<---	RWA	.087	.289	.134	.010
ChatGPT as a danger to humanity	<---	AI_CONCS	.199	.005	−.081	.060
ChatGPT as a danger to humanity	<---	Extraversion	.073	.334	−.168	<.001
ChatGPT as a learning tool	<---	Conscientiousness	.123	.017	−.020	.601
ChatGPT as a learning tool	<---	Extraversion	.144	.009	.028	.459

*Note.* Estimates presented in the model are standardized regression weights; AI_CONSC – interaction between AI literacy and Conscientiousness; AI_RWA – interaction between AI literacy and RWA; AI_EX – interaction between AI literacy and Extraversion; AI_NFC – interaction between AI literacy and NFC; AI_SDO – interaction between AI literacy and SDO; *** - statistically significant < .001; We did not examine whether results for men and women are statistically significantly different, this path model just allowed to see how the theoretical model works for men and women separately, as difference between ‘significant’ and ‘not significant’ is sometimes not statistically significant in itself (Gelman & Stern, 2006).

Table 3 depicts the significant paths in the model. We note here that reliability of certain measures in low (see section on instruments), which could have an effect on the stability of certain path coefficients. Thus, these results should be taken with caution. This table looks at how different factors are associated with people’s views about AI and ChatGPT, with a focus on gender differences.

#### Attitudes towards AI.

For women, AI literacy also predicts more positive attitudes, as well as SDO; unlike with men, AI literacy doesn’t ‘repair’ the image of AI, but it can predict a mixed opinionated image—high scores on both positive and negative attitudes towards AI. For men, AI literacy is associated with more positive attitudes while RWA is associated with lower scores on positivity, however, when interacting with AI literacy, RWA shows different effects on positive attitudes—it seems that in people with high RWA, AI literacy can make AI seem more positive.

#### ChatGPT as a personal assistant.

In women, this view of ChatGPT is predicted by openness, with higher openness predicting a stronger view of ChatGPT as a personal assistant. In women, this view of ChatGPT is also predicted by an interaction between AI literacy and SDO, AI literacy and NFC—all positively predicting this attitude. In men, this view of ChatGPT is predicted by an interaction between RWA and AI literacy, suggesting that those high on RWA and AI literacy understand GPT and how to use it to assist with work (i.e., for personal gain).

#### ChatGPT as a learning tool.

For women, only RWA predicts these beliefs, with higher RWA predicting a stronger view of ChatGPT as a learning tool*.* For men, conscientiousness and extraversion positively predict the belief that ChatGPT can be used as a learning tool. There was also an interaction between RWA and AI literacy, positively predicting this view of ChatGPT.

#### ChatGPT as a cognitive impairment risk.

For women, we found interaction effects of AI literacy on one hand, with extraversion and SDO on the other, negatively predicting this view of ChatGPT. In addition, another negative predictor for women was Emotionality. For men, positive predictors are RWA, SDO, and openness. For women, there is an interaction between AI literacy and RWA, positively predicting this view of ChatGPT.

#### ChatGPT as a danger to humanity.

In men, this is predicted positively by an interaction between AI and conscientiousness. In women, we found a negative main effect of extraversion, a positive main effect of RWA, and an interaction between RWA and AI literacy, with the interaction positively predicting this view of ChatGPT. In those with low RWA participants with high AI literacy show lower scores, and vice versa. In short, how people feel about AI and ChatGPT depends a lot on their personal traits and political views, and gender plays a role as well. We note that all interactions will be further explained after presenting their visualisation through graphs (see Fig 3 and Fig 4 for further details). Interaction effects in this study were calculated as a multiplicative interaction (multiplicative term)—where predictors and moderators are converted to Z scores, multiplied, and entered a path analysis as separate variables ([85]; applied in path analysis in, e.g., [86]). Visualisation of interactions was done using Jeremy Dawson slopes [Jeremy Dowson slopes: https://www.jeremydawson.co.uk/home].

**Fig 3:**
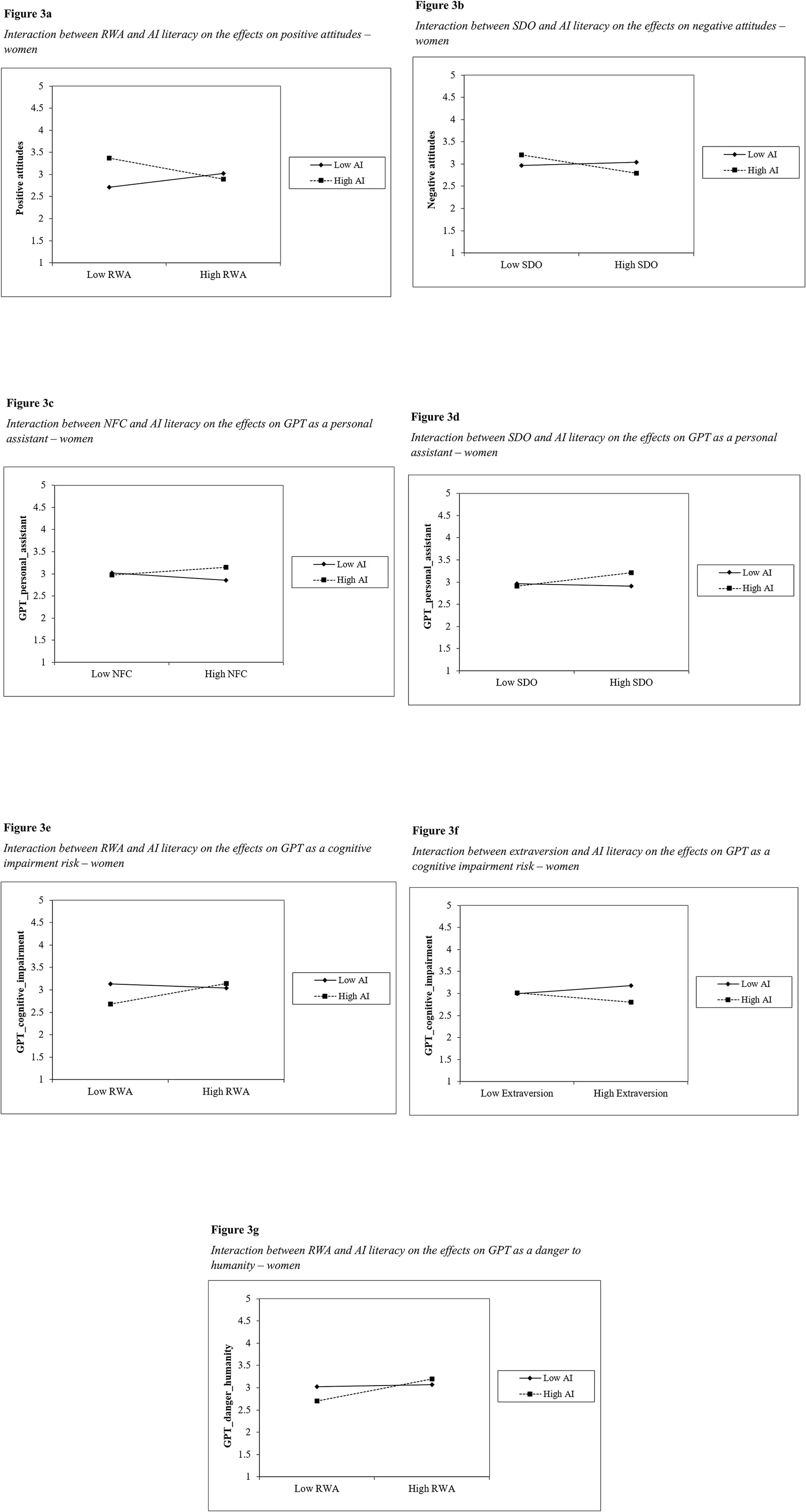
Interactions in the model—women.

**Fig 4:**
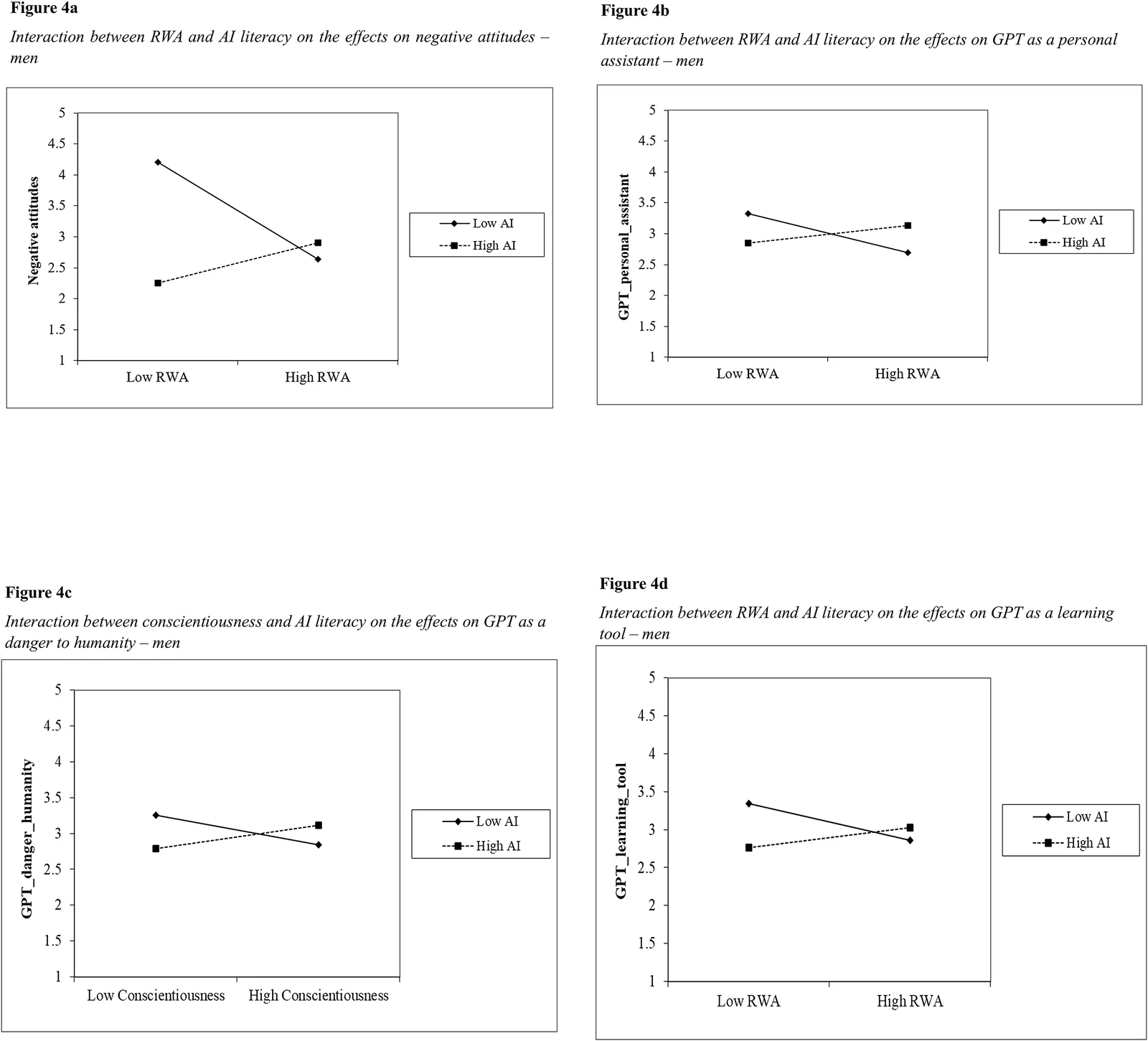
Interaction in the model—men.

Fig 3a showcases how individuals with low RWA and low AI literacy levels have less positive attitudes than those with low RWA and high AI literacy levels (who have the highest number of positive attitudes). When it comes to individuals with high RWA levels, those with low AI literacy levels have lower scores on positive attitudes in comparison to those with high AI literacy levels.

Fig 3b illustrates how individuals with low AI literacy and low SDO levels have less negative attitudes towards AI, while those with high AI literacy and low SDO levels have more negative attitudes towards technology. Data for individuals with high SDO levels shows opposite tendencies.

Fig 3c showcases how among individuals with low NFC scores, those low on AI literacy tend to see ChatGPT as a personal assistant to a greater extent, while those high on AI literacy tend to see ChatGPT in this manner to a lower extent. There are no differences among participants with high NFC scores.

Fig 3d illustrates how among participants with low SDO scores, those with low AI literacy levels tend to see ChatGPT as a personal assistant to a greater extent, while those with high AI literacy levels tend to see ChatGPT in this way to a lower extent. There are no differences among the participants with high SDO scores.

Fig 3e illustrates how among the participants with low RWA scores, those with low AI literacy levels tend to see ChatGPT as a cognitive impairment risk to a greater extent, while those with high AI literacy levels tend to see ChatGPT in this way to a lower extent. Among participants with high RWA levels, the differences subtly move in the opposite direction.

Fig 3f reveals that among participants with high extraversion levels, those with low AI literacy levels tend to see ChatGPT as a cognitive impairment risk to a greater extent, while those with high AI literacy levels tend to see ChatGPT in this way to a lower extent. There are no significant differences among participants with low extraversion levels.

Fig 3g showcases how among participants with low RWA levels, those with low AI literacy levels tend to see ChatGPT as a danger to humanity to a greater extent in comparison to those with high AI literacy levels. The situation is reversed in the case of participants with high RWA scores.

Fig 4a presents the interaction between RWA and AI literacy with regards to their effects on Negative attitudes in the men subsample. Those with low RWA and low AI literacy levels have the highest scores on negative attitudes, while those with high AI literacy and low RWA levels have the lowest scores. The results are the opposite for those with high RWA levels.

Fig 4b illustrates how among individuals with lower RWA levels participants with high AI literacy levels tend to view ChatGPT as a personal assistant at lower rates, in comparison to those with low AI literacy levels. Among those with high RWA levels, the situation is reversed.

Fig 4c showcases how among participants with low conscientiousness levels, those with low AI literacy levels tend to see ChatGPT as a danger to humanity to a greater extent in comparison to those with high AI literacy levels. The situation is reversed for participants with high conscientiousness levels.

Fig 4d illustrates that among participants with low RWA scores, those with low AI literacy levels tend to see ChatGPT as a learning tool to a greater extent in comparison to those with high AI literacy levels, with the situation being reversed for with high RWA scores.

In general, it seems that AI literacy gains its importance as a predictor through interaction with other variables rather than being important on its own. In summary, people’s views about AI and ChatGPT are predicted by both their political and social beliefs (RWA, SDO) and their knowledge of AI, with varying patterns across different personality traits like extraversion and need for cognitive closure, and gender and AI literacy as moderators, supporting our moderation-based hypotheses.

## Study 2

### Method

The aim of this study was to re-examine the role of gender, AI literacy, AI Attitudes, NFC, RWA, SDO, and the HEXACO personality traits in the prediction of attitudes towards ChatGPT on a sample of individuals belonging to a culture that is distinct from the one used in the first study, in this case, the German culture. Another aim was to examine differences between Serbian and German cultures on these variables.

### The sample

Participants filled in questionnaires via Google forms, they were recruited through social media (Facebook, Instagram, and LinkedIn) and through websites specializing in collecting data (SurveySwap and Prolific). Convenience sampling was used. The minimum sample size was determined through calculating a priori power for RMSEA (indicating minimum sample size of 94). Due to the model’s complexity, we were additionally led by rules of thumb for path analysis (5:1 or 10:1 participant per parameter; Westland, 2010). There were 115 participants from Germany, ages ranging from 18 to 71 (*M* = 29.85, *SD* = 9.64).. In the Serbian sample, there were 119 participants, ages ranging from 18 to 61 (*M* = 30.51, SD = 12.16) (see Table 4 and Table 5 for further sample description). Since the number of non-binary respondents was small, and a larger number of non-binary respondents was needed to include gender as a moderating variable, we excluded them from the analysis.. All respondents were given an informed consent form containing all information about the study. All participants agreed to participate in research.

**Table 4:** Percentage of participants per education level in Serbia and Germany in Study 2.

Education level	Serbia		Germany	
	*N*	%	*N*	%
High School Diploma	65	54.6	36	31.3
College Diploma	13	10.9	/	/
Studying towards Bachelor’s Diploma	7	5.9	/	/
Bachelor’s Diploma	26	21.8	58	50.4
Master/PhD	8	6.7	20	17.4
Missing	0	0	1	0.9

**Table 5:** Participants’ gender in Serbia and Germany in Study 2.

Gender	Serbia		Germany	
	*N*	%	*N*	%
Men	32	26.9	70	60.9
Women	85	71.4	42	36.5
Does not wish to tell	/	/	2	1.7
Non-binary	2	1.7	1	0.9

### Instruments

We used the same instruments as in Study 1. No specific adaptations for culture were done, since these instruments have already been used in these samples and in past studies (e.g., [5,87,88]), with one exception: the AI literacy measure was used and created for the German sample (see the details described in Study 1 and in the S1 File supplementary materials).

1. *The General Attitudes towards Artificial Intelligence Scale* (GAAIS; [5]). Both positive (α = .89, α = .85 on Serbian and German samples, respectively) and negative attitudes (α = .71, and α = .79, on Serbian and German samples, respectively) showed adequate reliability.
2. *HEXACO* was used to operationalize personality traits (24-item version; [75]). Internal consistency reliability varies between α = .35, to α = .56 in the Serbian sample, and between α = .31, to α = .56 in the German sample. The average inter-item correlation varies between .15 to .25 in both samples, which is considered an adequate level of reliability [73].
3. *Right-Wing-Authoritarianism* (RWA) had adequate internal consistency reliability (α = .86, α = .91, in Serbian and German samples respectively).
4. *Social dominance orientation* (SDO; [80]). Although its internal consistency reliability was low (α = .37, α = .58, in Serbian and German samples, respectively), the average inter-item correlation amounted to .18 and .32 in Serbian and German samples respectively, which suggests adequate reliability of the scale.
5. *Need for closure* (NFC; [82]). Internal consistency reliability was lower than adequate (α = .56, α = .59, in Serbian and German samples respectively). The average inter-item correlation was .19 and .23 in Serbian and German samples respectively, which suggests adequate reliability [73].
6. *AI literacy* measure (Weber et al., 2023).
7. To operationalize attitudes towards ChatGPT, we used a scale created in the pilot studies 1 and 2 (available in S1 File supplementary materials). ChatGPT as a personal assistant (α = .86, α = .78, in Serbian and German samples respectively), ChatGPT as a cognitive impairment risk (α = .83, α = .80, in Serbian and German samples respectively), ChatGPT as a danger to humanity (α = .73, α = .65, in Serbian and German samples respectively), and ChatGPT as a learning tool (α = .71, α = .70, in Serbian and German samples respectively).

### Procedure

The sample was collected and administered via Google Forms.

## Results

Descriptive statistics are shown in Supplementary materials (S1 File; Table 1D).

### Cross-cultural comparisons

We conducted *t*-tests to examine cross-cultural differences on all variables from the study. We note that these comparisons should be considered with caution, as measurement invariance across two compared cultures was not assessed. Future studies, done on larger and more representative samples. should address this.

Differences between cultures also appeared in the context of Positive attitudes towards AI (*t*(213.113) = −5.955, *p* < .001, CI 95% [−.75, −.377]) in favour of the participants from Germany (*M*difference = −.564; Cohen’s d = −.782).

When it comes to personality traits, differences were found for Conscientiousness (*t*(225.479) = 4.665, *p* < .001; CI 95% [.2, .493]) in favour of the participants from Serbia (*M*difference = .346; Cohen’s d = .617), and for Extraversion (*t*(225.394)= 3.657, *p* < .001), once again in favour of *t*he participants from Serbia (*M*difference = .31473; Cohen’s d = .482). Differences were also found for RWA (*t*(226.507) = 6.167, *p* < .001, CI 95% [.503, .976]) (*M*difference = .739; Cohen’s d = .814); SDO (*t*(226.999) = 2.768, *p* = .003, CI 95% [.134, .798]) (*M*difference = .466; Cohen’s d = .366); and for NFC (*t*(226.907) = 3.407, *p* < .001, CI 95% [.124, .463]), all in favour of the par*t*icipan*t*s from Serbia (*M*difference = .293; Cohen’s d = .45).

Differences were found in AI literacy (*t*(224.139) = −7.665, *p* < .001; CI 95% [−1.396, −.825]) in favour of German participants (*M*difference = --1.111; Cohen’s d = −1.01), and on all four dimensions of attitudes towards ChatGPT: ChatGPT as a cognitive impairment risk (*t*(226.709) = 5.667, *p* < .001, CI 95% [.491, 1.02]) in favour of *t*he participants from Serbia (*M*difference = .753; Cohen’s d = .749), ChatGPT as a danger to humanity (*t*(225.468) = 2.621, *p* = .005, CI 95% [.077, .542]) in favour of the par*t*icipants from Serbia (*M*difference = .309; Cohen’s d = .346), ChatGPT as a learning tool (*t*(226.988) = −3.119, *p* = .002, CI 95% [−.351, .113]) in favour of the par*t*icipants from Germany (*M*difference = −.351; Cohen’s d = −.412), ChatGPT as a personal assistant (*t*(223.265) = −2.284, *p* = .023, CI 95% [−.365, −.027]) in favour of the par*t*icipants from Germany (*M*difference = −.196; Cohen’s d = −.301).

We will proceed to examine differences between the two gender groups in each of the two cultures regarding attitudes towards AI in general and towards ChatGPT in particular. In the Serbian sample, differences between men and women were found only for AI literacy (*t*(51.805) = 2.674, *p* = .01, CI 95% [.165, 1.159]) in favour of men participants (*M*difference = .662; Cohen’s d = .577). In the German sample, differences between men and women were found for Positive attitudes towards AI in general (*t*(100.768) = 2.131, *p* = .046, CI 95% [.016, .452]) in favour of women (*M*difference = .234; Cohen’s d = .394), and on ChatGPT as a cognitive impairment risk (*t*(79.536)= −2.281, *p* = .025, CI 95% [−.84, −.057]) in favour of men (*M*difference = −.449; Cohen’s d = −.457).

These results show psychological differences in the two cultures—seen through differences in the role of personality traits. Even more, strong differences are noticed in attitudes towards AI, AI literacy and attitudes towards ChatGPT in particular, indicating that the German sample shows both higher AI literacy and more positive attitudes towards AI and ChatGPT. Gender differences show that more nuanced differences can be noticed in the German sample in comparison to the Serbian sample—suggesting that, in the Serbian sample, men and women are more equal in their attitudes towards and in their of knowledge of AI. After *t*-tests, we performed a path analysis through SEM.

### Path analysis

The path analyses conducted in this research include data-level modifications; thus, they should be considered as exploratory and future studies should aim to check them. Due to the small sample size in Study 2, we cannot claim that this analysis is a confirmatory one, this analysis is thus an exploratory way to check the model obtained on a larger Serbian sample in a German one. In the future, this model should be replicated in a larger representative sample for Germany. In addition, due to the small sample size, this path analysis is produced for the entire sample, not divided by gender group.

The Serbian model, produced without insignificant paths and using bootstrapping (1000 samples) did not achieve an adequate fit (Χ² = 646.917, *df* = 164, p < .001. Χ²/*df* = 3.945, GFI = .725, TLI = .479, IFI = .778, CFI = .757, RMSEA = .161, SRMR = .156), which is not surprising due to the fact that in Study 1, this model was checked separately for men and women, and in addition, on a larger sample. So, we proceeded to delete insignificant paths. The model with removed insignificant paths showed adequate fit (Χ² = 66.433, *df* = 51, p = .072. Χ²/*df* = 1.303, GFI = .931, TLI = .955, IFI = .98, CFI = .978, RMSEA = .052, SRMR = .081). The significant paths are presented in Table 7.

**Table 7:** Estimates of the path analysis in Study 2 (Serbian sample).

Criterion	Direction	Predictor	Estimate	*p*
Negative attitudes	<---	Emotionality	.324	.044
Negative attitudes	<---	AI_EMO	−.382	.025
Positive attitudes	<---	AI Literacy	.261	.009
ChatGPT as a personal assistant	<---	Positive attitudes	.563	.002
ChatGPT as a learning tool	<---	Positive attitudes	.578	.002
ChatGPT as a cognitive impairment risk	<---	Negative attitudes	.483	.002
ChatGPT as danger to humanity	<---	Negative attitudes	.560	.002
ChatGPT as a personal assistant	<---	Openness	.299	.006
ChatGPT as danger to humanity	<---	AI_OPEN	−.231	.036
ChatGPT as a personal assistant	<---	AI Literacy	.170	.002
ChatGPT as a cognitive impairment risk	<---	Agreeableness	.299	.005
ChatGPT as a cognitive impairment risk	<---	AI_OPEN	−.407	.004
ChatGPT as a cognitive impairment risk	<---	Openness	.225	.047

*Note.* Estimates presented in the table are standardized regression weights (bootstrapped); AI_OPEN—interaction between AI literacy and Openness to experience; AI_EMO—interaction between AI literacy and Emotionality.

Negative attitudes towards ChatGPT were significantly predicted by lower AI openness and lower emotionality. Positive attitudes were positively associated with AI literacy. In turn, positive attitudes strongly predicted perceiving ChatGPT as a helpful personal assistant or as a learning tool, while negative attitudes strongly predicted viewing ChatGPT as a cognitive impairment risk or as a danger to humanity. Individual differences also contributed: Openness and AI literacy positively predicted seeing ChatGPT as a personal assistant, whereas Agreeableness positively predicted perceiving it as a cognitive impairment risk. Interestingly, higher openness was associated with lower perception of ChatGPT as a cognitive impairment risk.

From the interaction graphs, we can see that for negative attitudes towards AI, participants with higher openness to experience have lower scores when they also have higher AI literacy, and higher scores when they have lower AI literacy. Opposite results are seen for participants with lower openness to experience (Fig 5a). Similar results are seen for ChatGPT as a danger to humanity (Fig 5b) and ChatGPT as a cognitive impairment risk (Fig 5c). For ChatGPT as a cognitive impairment risk, those differences are very small. This suggests that higher AI literacy and greater openness to experience are associated with less negative attitudes toward AI and ChatGPT.

**Fig 5:**
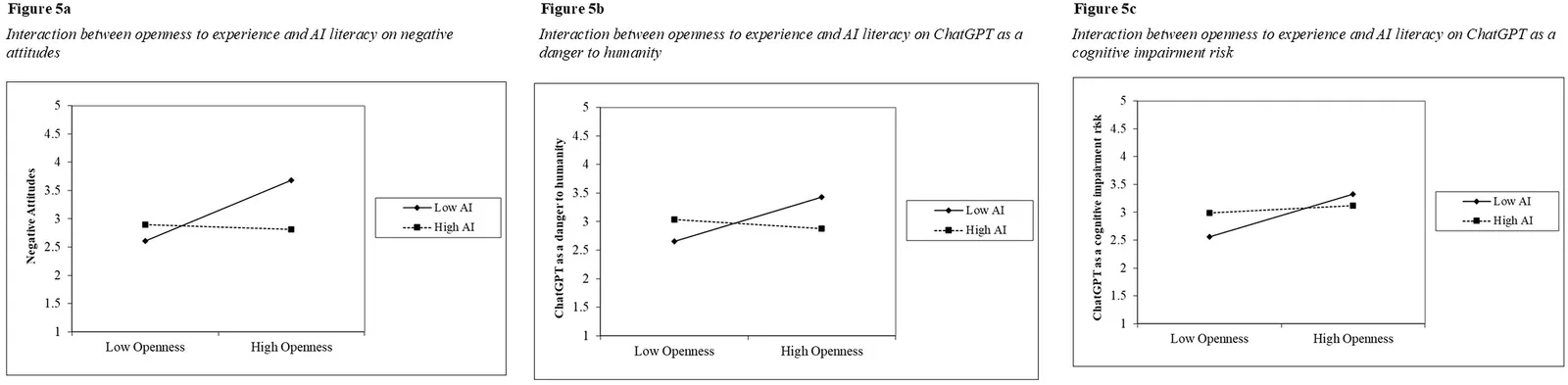
Interaction in the model – Serbian subsample.

The German model, produced without insignificant paths and using bootstrapping (1000 samples) showed an adequate fit (Χ² = 118.558, *df* = 93, p = .038. Χ²/*df* = 1.275, GFI = .906, TLI = .965, IFI = .982, CFI = .981, RMSEA .05, SRMR = .08). All fit indices achieved adequate levels. The significant paths are presented in Table 8. Exploratory model on German sample with gender as a moderator is presented in the supplementary materials (S1 File).

**Table 8:** Estimates of the path analysis in Study 2 (German sample).

Criterion	Direction	Predictor	Estimates	*p*
Negative attitudes	<---	RWA	.252	.022
ChatGPT as a cognitive impairment risk	<---	Negative attitudes	.412	.002
ChatGPT as danger to humanity	<---	Negative attitudes	.457	.002
ChatGPT as a personal assistant	<---	Positive attitudes	.579	.002
ChatGPT as a learning tool	<---	Positive attitudes	.606	.002
ChatGPT as a learning tool	<---	AI Literacy	−.207	.019
ChatGPT as danger to humanity	<---	AI Literacy	−.269	.002
ChatGPT as a learning tool	<---	Openness	.212	.018
ChatGPT as a learning tool	<---	RWA	.224	.005
ChatGPT as a personal assistant	<---	SDO	−.233	.018
ChatGPT as a cognitive impairment risk	<---	RWA	.215	.004

*Note.* Estimates presented in the table are standardized regression weights (bootstrapped).

We note here that the reliability of certain measures is low (see section on instruments), which could have an effect on the stability of the path coefficients. Thus, these results should be taken with caution.

From path model on the German sample, we firstly see that interaction effects are not as important as in Serbian sample—they do not predict attitudes towards AI nor attitudes towards ChatGPT. Of course, it is possible that this is due to the sample size/or gender as a confounding variable. When we consider gender (results presented in the S1 File supplementary materials), some interaction effects are significant (some only for men, some only for women in the sample). Hence, future research should collect a larger sample and check for interaction effects, dividing their sample by gender categories.

Participants’ negative attitudes towards ChatGPT were positively associated with Right-Wing Authoritarianism (RWA). Negative attitudes, in turn, predicted perceptions of ChatGPT as a cognitive impairment risk and as a potential danger to humanity, whereas positive attitudes predicted viewing ChatGPT as a useful personal assistant and learning tool. Higher AI literacy was associated with lower perceptions of ChatGPT as dangerous, though it was also slightly negatively related to its perceived utility as a learning tool. Individual differences also played a role: Openness and RWA positively predicted perceiving ChatGPT as a learning tool, Social Dominance Orientation (SDO) negatively predicted seeing it as a personal assistant, and RWA additionally predicted perceiving ChatGPT as a cognitive impairment risk.

## Discussion

The main aim of this article was to examine the potential predictors of attitudes towards ChatGPT. In the pilot studies, we first created the items and dimensions behind the attitudes towards ChatGPT. Subsequently, upon reviewing the literature, we established factors with potential connection to these attitudes. Finally, we tested six hypotheses, to be discussed in subsequent subsections.

### H1: Attitudes towards AI predict attitudes towards ChatGPT as particular tool

Positive attitudes towards AI predicted positively the positive dimensions of attitudes towards ChatGPT (ChatGPT as a learning tool, and ChatGPT as a personal assistant), while negative attitudes towards AI positively predicted negative attitudes towards ChatGPT (ChatGPT as a cognitive impairment risk and ChatGPT as a danger to humanity). This was expected. More positive attitudes towards AI in general are likely to be linked to more positive attitudes towards this specific tool. The interesting result here is that positive attitudes towards AI also predicted the presence of ChatGPT as a danger to humanity attitude (only for women in Serbian sample).

One possible explanation for this finding is that individuals with more positive attitudes towards AI possibly possess greater knowledge about ChatGPT, including an awareness of its potential risks. While our study measured AI literacy, it is important to note that familiarity with a system (e.g., through direct use or exposure) does not necessarily equate to actual knowledge about how it works (e.g.[89,90],). Previous research supports this distinction. For instance, Svikhnushina et al. [91] showed that familiarity with digital assistants can have both positive and negative effects on users’ trust and perceptions of usefulness. In other words, being familiar with a tool may enhance trust through perceived ease or comfort, but it may also reduce trust if that familiarity exposes users to limitations or risks. Given this nuance, future research should distinguish between trust, familiarity, and actual knowledge (i.e., AI literacy). Including all three may offer a more comprehensive understanding of how users form attitudes towards systems like ChatGPT.

### H2: Personality as predictor of ChatGPT-related attitudes

Personality traits play an important role in shaping attitudes towards ChatGPT, with slight differences between the two samples and depending on the respondent’s gender. Cultural differences will be discussed in detail later in the discussion.

In the women subsamples, ChatGPT as a danger to humanity is negatively predicted by Extraversion. The negative association with Extraversion contradicts previous research [5], which suggested that introverts are more prone to positive AI attitudes and have a more forgiving view of AI’s negative aspects. A possible explanation for the current result is that extraverted individuals, who are typically more socially engaged and enthusiastic about novel experiences [92], may perceive AI like ChatGPT as an exciting and socially beneficial innovation, rather than a threat.

For the ChatGPT as a learning tool dimension, Extraversion and Conscientiousness positively predicted such attitude in the men subsample (from Serbia). These results contrast with previous studies showing that high scores on these traits relate to less positive attitudes towards AI [5]. Although more nuanced scales like ours offer the potential to move beyond mere valence of an attitude to more specific content-related interpretations, there remains a need to examine how different individuals use ChatGPT to determine whether personal usage patterns explain these differences. Hence, hypotheses H2a (Extraversion predicts more negative attitudes towards AI and towards ChatGPT) and H2b (Conscientiousness predicts more negative attitudes towards AI and towards ChatGPT) could not be confirmed.

Although openness to experience is generally associated with positive attitudes towards AI [47], our findings suggest that this relationship could actually be context dependent. Specifically, openness showed mixed effects across outcomes (predicting both positive and negative attitudes), moderated by gender and cultural background. A possible explanation is that openness entails not just curiosity, but also a critical stance towards norms and structures [93], which may lead some individuals—especially those aware of AI’s potential to reinforce social inequalities—to adopt more critical attitudes. Thus, the impact of openness on AI attitudes cannot be assumed to be uniformly positive, but rather shaped by intersecting cultural and gendered experiences. Hence, H2c (Openness predicts more positive attitudes towards AI and towards ChatGPT) is only partially confirmed.

Emotionality negatively predicted the perception of ChatGPT as a cognitive impairment risk, but this effect was specific to the Serbian women subsample (Study 1). In Study 2, emotionality predicted negative attitudes towards AI (noting that results in Study 2 are conducted on a smaller subsample, hence there is a possibility of gender being a confounding variable). One possible explanation is that individuals high in emotionality—characterized by a tendency to experience anxiety, worry, and emotional sensitivity [48]—may be more inclined to use ChatGPT as a coping tool. This aligns with studies that show people tend to and can use digital tools for emotion regulation [94]. Future studies could explore how emotional traits shape not only risk perceptions but also the functions AI tools serve in users’ daily lives, especially across cultural and gender contexts. Hence, H2e (Emotionality predicts more negative attitudes towards AI and towards ChatGPT) could not be fully confirmed, showing that Emotionality can have a positive effect—but only for women and not in all cultures.

Agreeableness was negatively related to viewing ChatGPT as a learning tool in the German sample (S1 File supplementary data), while on the German sample as a whole (main manuscript), it was not a significant predictor. In the Serbian sample (whole sample, Study 2), agreeableness was also a predictor of seeing ChatGPT as a cognitive impairment risk. Agreeableness is usually connected to social motives and cooperation [95]. In addition, data shows that German participants are sceptical about usage of AI in education (ECO survey: High scepticism towards the use of AI in German schools, 2024 [ECO survey: High scepticism towards the use of AI in German schools. eco; 2024 Aug 6. Available from: https://international.eco.de/presse/eco-survey-great-scepticism-towards-the-use-of-ai-in-german-schools/]). Hence, a possible explanation is that agreeable individuals, who prioritize harmony and ethical considerations, may therefore be more sensitive to these societal concerns, leading to more negative attitudes towards AI in the German context. As such, hypothesis H2f (Agreeableness predicts more positive attitudes towards AI and towards ChatGPT) is not confirmed.

Finally, Honesty-Humility did not show any effect on ChatGPT attitudes, thus not offering sufficient support to our hypothesis H2d. Our general hypothesis that personality predicts attitudes towards ChatGPT was thus only partially confirmed. Our results are also not completely in line with previous studies, suggesting that personality relates somewhat differently to a particular AI tool like ChatGPT in comparison to AI technology in general. Further differences are noticed based on gender and culture as well. This finding calls for a re-examination of personality’s relationships with general and particular types of AI technologies, especially across groups and contexts.

### H3: Ideology and cognition as contributing factors to ChatGPT-related attitudes

RWA emerged as one of the strongest predictors of attitudes towards both AI and ChatGPT. In men, RWA predicts less positive and more negative attitudes towards AI and ChatGPT. In addition, in women, it also predicts the belief that ChatGPT is a learning tool (German subsample). One possible explanation for the latter result can be is that women generally tend to be more supportive towards authoritarian rules [19]. In addition, it goes in line with findings that people with high RWA endorse AI for some of its applications—those useful to them [6], but also findings that individuals with more conservative attitudes tend to be more sceptical of AI than liberals [50]. These results thus partially confirm our hypothesis H3a (Authoritarianism positively predicts attitudes towards AI and towards ChatGPT).

SDO positively predicted positive attitudes towards AI in the women subsample, but only in the Serbian sample. This contradicts both findings that SDO is not significantly related to AI attitudes [96], and studies indicating possible negative effects of SDO on such attitudes (e.g.[56],). These results disprove our hypothesis H3b (Social dominance orientation negatively predicts attitudes towards AI and towards ChatGPT). A possible explanation is that individuals high in SDO may develop positive attitudes towards AI when they perceive it as beneficial for personal gain, but only for women. This hypothesis should be further examined in future studies.

Our hypothesis that NFC negatively predicts attitudes towards AI and ChatGPT (H3c) as a main effect was disproved. However, we will see later that NFC has a conditional effect in interaction with AI literacy. Our general hypothesis that ideology and cognition are related to attitudes towards ChatGPT was partially confirmed, showcasing that although they might not be instinctively connected, political and social structure inclinations are important for the perception of AI tools.

### H4: AI literacy as crucial moderating factor between psychological aspects and attitudes towards ChatGPT

AI literacy was shown to be an important predictor of attitudes towards ChatGPT in German men, predicting both positive and negative attitudes, suggesting that AI literacy doesn’t necessarily predict positive attitudes but actually gives a balanced view of the tool. AI literacy also moderated relationship between some personality traits and attitudes towards both AI in general and ChatGPT in particular. For example, specifically in the Serbian sample, individuals high in Extraversion but low in AI literacy viewed ChatGPT as a cognitive impairment risk, whereas those with high AI literacy showed the opposite pattern. Previous studies suggested that extraversion is linked to more negative AI attitudes [5].Our findings indicate that AI literacy moderates this effect—extraversion alone does not determine AI attitudes, but its interaction with AI literacy does. One possible explanation is that extraversion is associated with impulsivity [97], and impulsivity is linked to less competent decision-making [98]. This could imply that extroverted individuals with high AI literacy engage in more critical thinking about ChatGPT, leading to nuanced attitudes. Regarding conscientiousness, the results could possibly mean that high-conscientiousness individuals [99] with high AI literacy [100] are more aware of ethical AI use. Future studies should explore these individual differences in ChatGPT usage. There were significant moderations in the German sample as well (S1 File supplementary), with AI literacy moderating negative effect of openness—open-minded people with higher AI literacy were more positive, and the same effect was found for agreeableness, showing that negative effect of personality traits can be lower in those with high AI literacy. In Study 2, in the full Serbian sample (not split by gender group), we see that in individuals with high openness, AI literacy is associated with lower negative attitudes towards AI and ChatGPT. Interestingly, AI literacy doesn’t moderate changes for all attitudes—for ChatGPT as a cognitive impairment risk, those effects are very small, which could suggest an inherent worry about ChatGPT’s effect on cognitive functioning.

Finally, our results suggest that individuals with low RWA and low AI literacy had the lowest positive attitudes towards AI and the highest negative attitudes towards AI. Among individuals with high RWA, those with lower AI literacy had more positive attitudes. These results could suggest that individuals with higher AI literacy may develop more balanced attitudes, acknowledging both AI’s potential and its risks [101]. In addition, AI literacy is also a standalone predictor (Study 2, full sample), where in the German sample, AI literacy predicts lower negative attitudes towards ChatGPT, and in the Serbian sample, it relates to higher positive attitudes towards AI. Based on this, we can conclude that our hypothesis about moderating effects of AI literacy (H4), is confirmed.

### H5: Gender as crucial moderating factor between psychological aspects and attitudes towards ChatGPT

*T*-tests showed gender differences in accordance with previous studies [10], confirming that men have more positive and less negative attitudes towards AI in general, and when it comes to ChatGPT in particular, women think ChatGPT represents a risk for cognitive impairment more than men do. Thus, these results confirm our hypothesis. Our structural model, divided by gender, additionally shows that positive attitudes for men and women are not always predicted by the same variables, and this is something future studies could take into account when examining gender differences in attitudes towards AI and tools like ChatGPT, as individual differences may act as confounding variables. For example, RWA predicted less positive attitudes towards AI and ChatGPT in men in both samples, but less so for women (especially when interacting with AI literacy). The results for men can be connected with the findings stating that conservatives tend to be more sceptical of AI [50] and for women with findings that individuals with high RWA have positive outlook towards some of the AI applications [6]. Women high in RWA might be more likely to view AI as protective or beneficial. In addition, SDO in Serbian women also predicted more positive attitudes, and mostly negative ones for men, suggesting that SDO may only relate to a more negative outlook towards AI [56] for men, and dependent on the culture. Due to their preference for social hierarchy [17] we could hypothesise that while high-SDO men could view AI as a levelling threat to their privileged positions, high-SDO women might perceive AI as an opportunity to challenge or renegotiate those hierarchies within constrained systems.

Apart from ideology, gender was found to be significant moderator of the effects of personality traits as well. For example, while extraversion and openness are significant predictors for both men and women, they predict different attitudes. Agreeableness seems relevant only for women, and conscientiousness seems more important for Serbian men. The reasons for such differences could lie in underlying gender differences in personality traits [18]. Thus, our hypothesis (H5: Gender is a significant moderator in the relationship between Personality traits, Ideology variables (SDO, RWA), Cognition (NFC), and attitudes towards AI and ChatGPT, leading to different relationships between predictor and outcome variables for men versus women) is confirmed.

### H6: Cross-cultural differences in attitudes towards ChatGPT

In this study, we first examined the differences between Serbian and German participants. We found that AI literacy is higher in German participants, which was expected since Germany is more of an “AI early adopter” in comparison to Serbia. The higher AI literacy in Germany appears to translate into more positive attitudes towards AI and ChatGPT, suggesting that familiarity and trust in technological advancement [11] may be associated with lower scepticism, whereas in Serbia, the relative novelty of such technology may trigger more caution or resistance. Differences were also found regarding some personality traits—conscientiousness and extraversion—with both being higher in participants from Serbia. According to the results of the multidimensional scaling of personality, Serbs are more impulsive, warm, active, cheerful, imaginative, liberal, trusting, competent, organized, and self-disciplined in comparison to Germans [102]. This is in accordance with our results regarding the above-mentioned two personality traits. Differences between cultures also appear in RWA, SDO, and NFC, all in favour of participants from Serbia. This could be explained by results related to Hofstede’s dimension power distance, which is quite high in Serbia [Country comparison tool. Hofstede Insights. Available from: https://www.hofstede-insights.com/country-comparison-tool?countries=serbia].

A possible explanation for our results regarding gender could lie in cultural socialisation and gender role expectations. In countries like Germany, which are characterized by lower power distance, higher individualism, and more egalitarian gender norms [70,103], both men and women are exposed to relatively similar opportunities and challenges in the technological sphere. However, women in such contexts may still engage in more critical assessments of AI due to gendered socialisation processes that emphasize careful risk evaluation and empathy [104]. In contrast, in Serbia, where cultural traditions may place greater emphasis on hierarchical structures and stricter gender roles (as reflected in a higher power distance^5^, gendered attitudes towards technology might be less ambivalent, with societal norms potentially steering both men and women towards more uniform perceptions of technological risks and benefits. The role of these mechanisms should be empirically verified in future studies.

Another mechanism involves the interplay between personality traits and cultural context. Namely, in Study 2, we can see that different traits predict attitudes towards AI and ChatGPT. Through our exploratory analysis of gender-based paths on the German sample (see S1 File supplementary materials), we additionally see a more nuanced pattern among women, where we see that traits like the NFC can become significant predictors. This could potentially suggest that in cultures with advanced technological infrastructure and greater digital literacy, women may develop a heightened sensitivity to information processing and decision-making under uncertainty [105]. In contrast, the Serbian context—with its relatively low AI literacy and more traditional gender role expectations—might not stimulate the same cognitive engagement, leading to less pronounced gender-specific predictors. Furthermore, the differential effects of factors like RWA on AI attitudes—wherein RWA negatively predicts positive AI attitudes in men across both cultures but shows divergent effects in women—could be a reflection of how cultural and gender norms interact to shape perceptions of authority and risk [50]. The hypotheses and possible explanations we gave in this part should be carefully and empirically checked in future studies. Nevertheless, these findings highlight that both cultural context and gender socialization processes could create unique environments where personality traits express themselves differently, ultimately relating to attitudes towards AI and technologies like ChatGPT.

In conclusion, our last hypothesis (H6: There are significant differences in attitudes towards AI and ChatGPT between the two compared cultures. In particular, our expectation that Germans tend to be more positively oriented towards AI and ChatGPT than Serbs) was confirmed.

### Limitations and Future studies

Despite the valuable insights provided by this study, several limitations should be acknowledged. First, the study relies on self-report measures, which are subject to social desirability bias and individual differences in self-perception. Participants may have responded in ways they perceived as socially acceptable rather than reflecting their actual attitudes or behaviours towards ChatGPT. Future research should consider incorporating behavioural measures or implicit attitude tests to complement self-reported data.

Second, while we examined cross-cultural differences by comparing German and Serbian samples, these results may not be generalizable to broader populations. Cultural differences in AI literacy, education systems, and exposure to technology could have an effect on attitudes towards ChatGPT in ways not fully captured by our study. Future research should aim for more diverse and representative samples, including participants from other countries and cultural contexts.

Third, the study’s sample size, particularly in Study 2, is relatively small compared to Study 1, which may limit statistical power and the robustness of certain findings. While we detected significant relationships, a larger sample would provide greater confidence in the stability of these effects. Future research should aim to replicate and extend these findings with larger and more balanced samples.

Fourth, the study primarily focuses on individual psychological predictors of ChatGPT attitudes, such as personality traits and ideological orientations. However, external factors, such as media exposure, prior experiences with AI, and professional background, may also play crucial roles in shaping attitudes. Future research should explore the interplay between personal characteristics and environmental influences.

Fifth, while we examined AI literacy as a moderator, the measure of AI literacy was relatively broad and did not assess domain-specific expertise. Individuals with higher AI literacy may have varying degrees of knowledge across different AI applications, which could affect their attitudes differently. More refined measures of AI literacy, including domain-specific knowledge, could provide deeper insights.

Further, although we found some gender differences in attitudes towards ChatGPT, our study did not explore potential underlying mechanisms, such as socialization processes or gendered experiences with technology. Future studies should investigate why these differences emerge and whether they are stable across different AI applications. An additional limitation was the fact that we included only men and women, due to a low number of participants of other gender identities, hence this is also something that should be examined in future research.

Further, future studies should account for real and objective risks and benefits of tools like ChatGPT and account them as confounding variable—as we don’t know do people’s attitudes aling with objective risks and benefits.

Lastly, future research should also account for variability in the purposes for which ChatGPT is used and how often. Despite these limitations, our study contributes to the growing body of research on AI-related attitudes, offering novel insights into how personality, ideology, cognition, and AI literacy shape perceptions of ChatGPT. Future research should build upon these findings to further refine our understanding of human-AI interactions.

### Practical contributions

The findings have several important practical applications. First, they underscore the critical importance of AI literacy and reveal notable cultural and gender differences in this area. These differences highlight the need for targeted educational strategies to address inequalities in knowledge, as this knowledge appears to play a crucial role in shaping individuals’ attitudes towards AI, particularly ChatGPT. As such, these results offer valuable insights for initiatives aimed at promoting AI knowledge and digital competencies, emphasizing the need for inclusive and culturally aware educational approaches. This is particularly relevant if we review newer studies which show that attitudes toward AI, and engagement with it, can go through different evolution stages through its usage (e.g., usage by young people during university-related transition; [106]), from limited engagement, through experimentation with various tools followed by recognising both benefits and drawbacks, to more regulated use.

Second, the results demonstrate that personality and ideology are significant factors, but their impact varies across cultures and gender groups. For instance, extraversion seems to have a stronger association with these attitudes for men, while emotionality plays a greater role for women. This suggests that individuals make decisions regarding ChatGPT based on different psychological traits, which could inform more personalized and culturally sensitive strategies. Training programs aimed at spreading AI knowledge should thus consider personality traits to better tailor their approaches.

Additionally, these results highlight the importance of considering psychological factors in user engagement strategies for AI technologies. By understanding the varied psychological traits that can have an effect on people’s interactions with AI, businesses and educational institutions can better design platforms, tools, and content that cater to individual preferences and enhance user experience. Tailoring AI tools to specific psychological profiles could lead to higher engagement, more effective learning experiences, and broader adoption of AI technologies.

Finally, the scale we present here measuring attitudes towards ChatGPT can be easily adapted to measure attitudes towards LLMs and thus used in numerous studies. This is particularly useful as some studies show that attitudes towards AI are relatively stable in different time periods (e.g.[107,108],). Overall, these results offer a framework for developing more individualized approaches to AI education, helping to reduce inequalities and enhance digital competencies across different cultural and gender groups.

## Conclusions

In general, this study sheds new light on predictors of attitudes towards AI in general and ChatGPT in particular. This study highlights the critical role of AI literacy, cultural and gender differences, and psychological traits in shaping individuals’ attitudes towards ChatGPT. It demonstrates that AI knowledge is a key predictor of these attitudes and calls for targeted educational strategies to address inequalities in AI literacy. Additionally, the study emphasizes the importance of considering personality and ideology when designing tailored interventions, as these factors are associated with decision-making in different cultural and gender contexts. Overall, the findings provide actionable insights for educational, marketing, and policy initiatives aimed at fostering digital competencies and reducing disparities in AI knowledge.

## Supporting information

S1 File Supplementary materials. (DOCX)
