# Supplementary material for: Cognitive impairment and danger to humanity, or personal assistant and learning tool? Exploring attitudes towards ChatGPT and their predictors
Source: PLoS One. 2026 Oct 8;21(10):e0341693. doi: 10.1371/journal.pone.0341693 (PMC13648967; doi:10.1371/journal.pone.0341693)
Supplement: Supplementary file 1 [file pone.0341693.s001.docx]

# **Supplementary material**

## **A. Pilot Study 1**

*A.1. Aim of the Study*

The aim of this pilot study was to explore the different facets of attitudes towards ChatGPT.

*A.2. The Sample and Procedure*

The sample consisted of 16 individuals from Serbia (68.8% women; age ranged from 21 to 48, *M* = 32.37, *SD* = 7.79) who rated several close-ended items concerning ChatGPT and gave their own open-ended answers to questions regarding the positive and negative aspects of this tool.

**Table 1A**

*Education in sample for pilot 1*

| Level | N | % |
| --- | --- | --- |
| High School | 3 | 18.8 |
| Towards finishing bachelor studies | 1 | 6.3 |
| Bachelors | 2 | 12.5 |
| Master | 10 | 62.5 |
| Total | 16 | 100.0 |

Closed-ended items were constructed after an internet search (blogs, and forums, e.g.: <https://tesseract.academy/chatgpt-report-attitudes-and-predictions-for-the-future/>) regarding attitudes towards this tool. The rest of the items were constructed based on open-ended answers from participants. The open-ended questions were: “What are the good sides of ChatGPT?”, “For what purpose can it be used?”, “Are there any bad sides of ChatGPT?”, and “Are there any dangers of ChatGPT?”. Based on these analyses, we drew 40 items in total. All items are presented in Table 1A.

**Table 2A**

*Items created in the Pilot 1*

ChatGPT…

| **Positive aspects** | **Negative aspects** |
| --- | --- |
| **Items created based on internet search** | |
| 1. can be very useful if regulated | 1. shows that humans can be replaced by artificial intelligence |
| 2. can be used to stimulate innovation in organizations | 2. will be used by students to write their term papers which will negatively impact education |
| 3. can be used to stimulate innovation in education | 3. will make some jobs redundant |
| 4. can improve efficiency in the workplace | 4. is very dangerous |
| 5. can improve efficiency in the education | 5. can be a weapon of mass destruction |
| 6. can help with repetitive tasks | 6. can help people to commit crimes which will lead to a higher crime rate |
| 7. can make work easier |  |
| **Items created based on open-ended answers in pilot study** | |
| 1. It can be helpful in information collection | 1. It can be misused |
| 2. It can be helpful in learning about fields other than your own | 2. You can reach information on how to harm yourself or others more easily |
| 3. It can help to learn a new language | 3. It is superficial |
| 4. It will remove unnecessary steps in some jobs | 4. It can break down when you need it the most |
| 5. It can be helpful to translate texts | 5. The human brain will atrophy from the lack of usage |
| 6. It can be helpful to finish up administrative stuff | 6. It will help present creative content as your own |
| 7. It is a very fast encyclopedia | 7. We will be taking credit for the solutions this tool arrived to |
| 8. It helps foster creativity | 8. It threatens humans because it learns, and it can become smarter than us |
| 9. It helps with some types of jobs like content writing | 9. Cognitive functions in children can develop more slowly |
| 10. It helpq ro explain stuff step by step | 10. The information it gives us is not accurate |
| 11. It is there to help us when we lack the inspiration | 11. Critical thinking can become extinct |
| 12. It is there to help us with formal speech | 12. It will foster laziness |
| 13. It is innovative |  |
| 14. It is neutral, it does not take sides |  |
| 15. It responds very fast |  |

In the second pilot study, we further analyzed the items constructed in this phase and found the dimensions underlying them.

## **B. Pilot Study 2**

*B.1. Aim of the Study*

The aim of this study was to explore the items constructed in the first study, and to find dimensions underlying the attitudes towards ChatGPT.

*B.2. Method*

*B.2.1. Sample*

Fifty respondents participated in the second pilot study. This sample size was chosen due to previous studies suggesting a minimum of 50 respondents for factor analysis (de Winter et al., 2009). A convenience sample was used. The age ranged from 19 to 60 years (*M* = 28.42, *SD* = 8.19). Fifty-two percent of respondents were women and 48% were men. Survey was available for participants in English and Serbian, 44% of participants were from Serbia.

Table 2B

*Education in sample for pilot 2*

| Level | N | % |
| --- | --- | --- |
| High School | 11 | 22.0 |
| Towards finishing bachelor studies | 4 | 8.0 |
| Bachelors | 15 | 30.0 |
| Master | 13 | 26.0 |
| PhD graduate/PhD candidate | 7 | 14.0 |
| Total | 50 | 100.0 |

*B.2.2. Instruments*

1. Socio-demographic questions, regarding gender, age, and education level;

2. Items constructed in the first pilot study regarding the attitudes towards ChatGPT.

3. AI literacy scale (Pinksy, 2023). This is an objective measure of AI literacy, consisting of 16 theoretical questions, 8 for lay individuals and 8 for experts, rounded on four dimensions: Socio User AI Literacy, Socio Creator/Evaluator AI Literacy, Technical User AI Literacy, and Technical Creator/Evaluator AI Literacy, with “User” dimensions being for lay individuals. Out of the 8 items for lay individuals, we chose 4 items most suitable for the lay participants. In our first attempt to conduct research, our respondents were presented with all items, but some respondents declined filling in the questionnaires due to the difficulty of these items. We were led by Bloom’s taxonomy when creating this scale and by their example when selecting items – we selected items that relied on remembering rather than on more complex forms of knowledge. In the end, we had 2 items per scale. A possible reason for the decision not to respond to these items can be found in the fact that this scale was created for German participants, with somewhat higher AI literacy levels when compared to Serbian participants, hence the Serbian sample found these items to be quite hard.

*B.2.3. Procedure*

The sample was collected online, using Google forms and social networks. Respondents answered 40 questions regarding the attitudes concerning ChatGPT (see Table 1A) constructed on the basis of the first pilot study.

*B.2.4. Data Analysis*

The data was analyzed using SPSS and JASP. We conducted exploratory factor analysis (EFA) to explore dimensions behind the items presenting attitudes towards ChatGPT, reliability analysis, and confirmatory factor analysis (CFA) to confirm the obtained dimensional structure. We also checked the correlation between AI literacy and attitudes towards ChatGPT.

*B.3. Results*

In this section of the paper, we will present the results of the exploratory factor analysis on the pilot sample, as well as the results obtained by the reliability analysis.

Bartlett’s test was statistically significant (Χ²(780) = 1474.77, *p* < .001), suggesting that we can proceed with the factor analysis. We first found a two-factor solution according to the parallel analysis, we named the first factor “Positive Attitudes” and the second “Negative Attitudes”. The explained percentage of variance was low, however (38%). Table 2B shows a two-factor solution.

**Table 2B**

*Factor loading of the two-factor solution*

| **Item** | **Positive atttudes** | **Negative attitudes** | **Uniqueness** |
| --- | --- | --- | --- |
| 1. It can be very useful if regulated | 0.452 |  | 0.777 |
| 2. It shows that humans can be replaced by artificial intelligence | 0.573 | 0.509 | 0.471 |
| 3. It can be used to stimulate innovation in organizations | 0.831 |  | 0.230 |
| 4. It will be used by students to write their term papers which will negatively impact education |  | 0.399 | 0.831 |
| 5. It can be used to stimulate innovation in education | 0.720 |  | 0.360 |
| 6. It will make some jobs redundant |  | 0.555 | 0.656 |
| 7. It can improve efficiency in the workplace | 0.694 | -0.360 | 0.338 |
| 8. It is very dangerous |  | 0.594 | 0.579 |
| 9. It can improve efficiency in the education | 0.642 |  | 0.478 |
| 10. It can be a weapon of mass destruction |  | 0.536 | 0.669 |
| 11. It can help with repetitive tasks | 0.782 |  | 0.343 |
| 12. It can help people to commit crimes which will lead to a higher crime rate |  | 0.514 | 0.737 |
| 13. It can make work easier | 0.849 |  | 0.266 |
| 14. It can be helpful in information collection | 0.702 |  | 0.512 |
| 15. It can be misused |  | 0.425 | 0.821 |
| 16. It can be helpful in learning about fields other then your own | 0.762 |  | 0.422 |
| 17. You can reach information on how to harm yourself or others more easily |  | 0.542 | 0.707 |
| 18. It can help to learn a new language | 0.314 |  | 0.858 |
| 19. It is superficial |  |  | 0.907 |
| 20. It will remove unnecessary steps in some jobs | 0.749 |  | 0.437 |
| 21. It can break down when you need it the most |  |  | 0.867 |
| 22. It can be used to translate texts |  |  | 0.967 |
| 23. The human brain will atrophy from the lack of usage |  | 0.606 | 0.571 |
| 24. It can be used to finish up administrative stuff | 0.645 |  | 0.588 |
| **25. It will help present creative content as your own** | 0.531 | 0.372 | 0.619 |
| 26. It is a very fast encyclopedia | 0.636 |  | 0.585 |
| **27. People will take credit for the solutions this tool arrived to** | 0.742 |  | 0.407 |
| 28. It helps foster creativity | 0.316 | -0.350 | 0.755 |
| 29. It threatens humans because it learns, and it can become smarter than us |  | 0.689 | 0.497 |
| 30. It helps with some types of jobs like content writing | 0.666 |  | 0.561 |
| 31. Cognitive functions in children can develop more slowly |  | 0.683 | 0.524 |
| 32. It can explain stuff step by step | 0.806 |  | 0.309 |
| 33. The information it gives us is not accurate |  |  | 0.909 |
| 34. It is there to help us when we lack the inspiration | 0.417 |  | 0.810 |
| 35. Critical thinking can become extinct |  | 0.646 | 0.581 |
| 36. It can be used to help us with formal speech | 0.502 |  | 0.698 |
| 37. It will foster laziness |  | 0.596 | 0.643 |
| 38. It is innovative |  |  | 0.930 |
| 39. It is neutral, it does not take sides |  |  | 0.949 |
| 40. It responds very fast | 0.662 |  | 0.565 |

*Note*. Bolded items can be reversly coded.

After this, we conducted an internal consistency reliability analysis.

**Table 3B**

*Reliability of the two-factor solution*

| Dimension | ω | α |
| --- | --- | --- |
| Positive Attitudes | 0.929 | 0.928 |
| Negative Attitudes | 0.847 | 0.847 |

Table 3B shows that both dimensions possess adequate reliability.

Although we obtained an adequate factor structure, we conducted another EFA, with maximum likelihood and Guttman-Kayser criteria to check whether we could find dimensions beyond the mere valenced distinction between positive and negative attituders, and to reduce the number of items by deleting inadequate ones. Another reason for conducting an additional analysis is the explained percentage of variance – which was relatively low in the first two-factor solution.

The second EFA accounted for 50.9% of the total variance. However, this analysis resulted in a solution with 5 factors where one of them had only two items. Thus, we used a theoretical number of factors and fixed the factor solution to four factors. Bartlett’s test was statistically significant (Χ²(780) = 1474.77, *p* < .001), meaning that the conditions for EFA are met, hence we proceeded with the analysis.

Table 4B contains the final factor loadings.

**Table 4B**

*Factor characteristics of the four-factor solution*

| Factor | Sum of Squares | Proportion of variance | Cumulative proportion |
| --- | --- | --- | --- |
| Factor 1 | 8.904 | 0.223 | 0.223 |
| Factor 2 | 3.803 | 0.095 | 0.318 |
| Factor 3 | 3.408 | 0.085 | 0.403 |
| Factor 4 | 2.568 | 0.064 | 0.467 |

Table 4B suggests that all factors can account for 46.7% of the total variance. Next, we proceeded with a CFA in accordance with the results from EFA. The first results from CFA suggest that the model does not fit well with the data (Χ² = 1346.793, *df* = 435, *p* < .001; CFI = .729; NNFI = .704; IFI = .739; RMSEA = .111; SRMR = .124). Due to this, we deleted items suggested by modification indices and correlated a few residuals. The corrected model showed an adequate fit to the data (Χ² = 149.863, *df* = 140, *p* = .269; CFI = .978; NNFI = .973; IFI = .979; RMSEA = .038; SRMR = .092), except for the SRMR which was slightly higher than the border level.

Table 5B reveals that only two items have a factor loading below .50. Hence, we decided to remove them from the scale. After removing them, we repeated the CFA to derive new fit indices. The results from the second CFA suggest that the model has an adequate fit to the data (Χ² = 100.157, *df* = 107, *p* = .667; CFI = 1.000; NNFI = 1.020; IFI = 1.015; RMSEA = .000; SRMR = .083).

**Table 5B**

*Factor loadings*

| Factor | Item | Estimate |
| --- | --- | --- |
| Factor 1 | GPT3 | 0.647 |
|  | GPT11 | 0.713 |
|  | GPT13 | 0.642 |
|  | GPT14 | 0.639 |
|  | GPT20 | 0.580 |
|  | GPT24 | 0.526 |
|  | GPT26 | 0.524 |
| Factor 2 | **GPT4** | **0.436** |
|  | GPT23 | 0.973 |
|  | GPT31 | 0.917 |
|  | GPT35 | 0.864 |
|  | GPT37 | 0.974 |
| Factor 3 | GPT12 | 0.580 |
|  | GPT17 | 0.787 |
|  | GPT29 | 0.908 |
|  | **GPT39** | **0.238** |
| Factor 4 | GPT5 | 0.825 |
|  | GPT9 | 0.754 |
|  | GPT28 | 0.613 |

**Table 5B**

*The final version of the scale based on CFA with four factors*

| Factor | Item number | Item content |
| --- | --- | --- |
| Factor 1: Chat-GPT as a personal assistant | GPT3 | 3. It can be used to stimulate innovation in organizations |
|  | GPT11 | 11. It can help with repetitive tasks |
|  | GPT13 | 13. It can make work easier |
|  | GPT14 | 14. It can be helpful in information collection |
|  | GPT20 | 20. It will remove unnecessary steps in some jobs |
|  | GPT24 | 24. It can help to finish up administrative staff |
|  | GPT26 | 26. It is a very fast encyclopedia |
| Factor 4: Chat-GPT as a learning tool | GPT5 | 5. It can be used to stimulate innovation in education |
|  | GPT9 | 9. It can improve efficiency in the education |
|  | GPT28 | 28. It helps foster creativity |
| Factor 2: Chat-GPT as cognitive impairment risk | GPT23 | 23. The human brain will atrophy from the lack of usage |
|  | GPT31 | 31. Cognitive functions in children can develop more slowly |
|  | GPT35 | 35. Critical thinking can become extinct |
|  | GPT37 | 37. It will foster laziness |
| Factor 3: Chat-GPT as a danger to humanity | GPT12 | 12. It can help people to commit crimes which will lead to a higher crime rate |
|  | GPT17 | 17. You can reach information on how to harm yourself or others more easily |
|  | GPT29 | 29. It threatens humans because it learns, and it can become smarter than us |

The first factor is mostly saturated by items referring to some form of effectiveness and personal assistance; hence it was named “Chat-GPT as a personal assistant”. The second factor contains items that suggest that ChatGPT will lead to a lack of critical thinking and cognitive development; hence it was named “Chat-GPT as cognitive impairment risk”. The third factor refers to items connected to the possibility of misuse and associated dangers, in that context, the third factor was named “Chat-GPT as a danger to humanity”. Finally, the fourth factor refers to education and self-improvement, and thus we named it “Chat-GPT as a learning tool”.

**Figure 1B**

*Final plot of the model*

Figure 1B shows the final model as analyzed in the CFA.

After the CFA, we also conducted a reliability analysis of the four-factor solution.

**Table 6B**

*Reliability of the four-factor solution*

| Dimension | ω | α |
| --- | --- | --- |
| Chat-GPT as a personal assistant | 0.889 | 0.891 |
| Chat-GPT as a learning tool | 0.788 | 0.776 |
| Chat-GPT as cognitive impairment risk | 0.859 | 0.858 |
| Chat-GPT as a danger to humanity | 0.714 | 0.694 |

From Table 6B, we can conclude that all dimensions are reliable. Since previous studies suggested a positive correlation between AI literacy and attitudes towards AI, we wanted to check the correlation between AI literacy and the newly constructed ChatGPT-attitude dimensions. The correlation between AI literacy and ChatGPT attitudes is shown in the Table 7B, along with descriptive statistics of AI literacy and newly acquired ChatGPT attitude dimensions.

**Table 7B**

*Descriptive statistics*

| Variables | *Min* | *Max* | *M* | *SD* |
| --- | --- | --- | --- | --- |
| AI Literacy | 0.00 | 4.00 | 1.88 | 1.72 |
| ChatGPT as a personal assistant | 1.00 | 5.00 | 4.27 | 0.66 |
| ChatGPT as a learning tool | 1.00 | 5.00 | 3.88 | 0.85 |
| ChatGPT as a cognitive impairment risk | 1.00 | 5.00 | 2.96 | 1.02 |
| ChatGPT as a danger to humanity | 1.00 | 4.67 | 2.79 | 0.92 |

**Table 8B**

*Correlation between AI literacy and ChatGPT attitudes*

| Variables | AI Literacy |
| --- | --- |
| ChatGPT as a personal assistant | .169 |
| ChatGPT as a learning tool | .381^**^ |
| ChatGPT as a cognitive impairment risk | -.211 |
| ChatGPT as a danger to humanity | -.111 |

From the previous table, we can see that only one correlation coefficient is significant. Although, we should bear in mind that this pilot sample is small, and that lack of significance can be due to this fact.

**Table 9B**

*Correlation between ChatGPT attitudes*

| **Correlations** | | ChatGPT as a personal assistant | ChatGPT as a learning tool | ChatGPT as a cognitive impairment risk | ChatGPT as a danger to humanity |
| --- | --- | --- | --- | --- | --- |
| ChatGPT as a personal assistant | Pearson Correlation | 1 | .609^***^ | -.133 | .041 |
| ChatGPT as a learning tool | Pearson Correlation | .609^***^ | 1 | -.334^*^ | -.216 |
| ChatGPT as a cognitive impairment risk | Pearson Correlation | -.133 | -.334^*^ | 1 | .421^**^ |
| ChatGPT as a danger to humanity | Pearson Correlation | .041 | -.216 | .421^**^ | 1 |

## **C. Study 1**

**Table 1C**

*Descriptive statistics from Study 1 (Serbian sample)*

| Variables | *Min* | *Max* | *M* | *SD* |
| --- | --- | --- | --- | --- |
| AI Literacy | 0.00 | 4.00 | 1.49 | 1.06 |
| NFC | 1.60 | 5.00 | 3.76 | 0.64 |
| SDO | 1.00 | 7.00 | 3.81 | 1.29 |
| RWA | 1.00 | 5.00 | 2.89 | 0.89 |
| Openness | 1.25 | 5.00 | 3.78 | 0.70 |
| Conscientiousness | 1.50 | 5.00 | 3.60 | 0.74 |
| Agreeableness | 1.00 | 4.75 | 2.98 | 0.67 |
| Extraversion | 1.50 | 5.00 | 3.90 | 0.74 |
| Emotionality | 1.00 | 5.00 | 2.93 | 0.79 |
| Honesty-Humility | 1.00 | 5.00 | 3.65 | 0.87 |
| Positive attitudes | 1.00 | 5.00 | 3.05 | 0.83 |
| Negative attitudes | 1.00 | 5.00 | 2.99 | 0.73 |
| GPT as a personal assistant | 1.00 | 5.00 | 3.81 | 0.79 |
| GPT as a cognitive impairment | 1.00 | 5.00 | 3.61 | 1.04 |
| GPT as a danger humanity | 1.00 | 5.00 | 3.09 | 0.94 |
| GPT as a learning tool | 1.00 | 5.00 | 3.31 | 0.94 |

**Table 2C**

*Correlations between variables in the study*

|  | ChatGPT as a personal assistant | ChatGPT as a cognitive impairment risk | ChatGPT as a danger to humanity | ChatGPT as a learning tool | |
| --- | --- | --- | --- | --- | --- |
| AI Literacy | .215*** | -.181*** | -.153*** | .126** |  |
| Gender | -.033 | .169*** | .041 | -.055 |  |
| Openness | .189*** | .045 | .001 | .074 |  |
| Conscientiousness | .009 | -.028 | .031 | .043 |  |
| Agreeableness | -.022 | -.090 | -.001 | .048 |  |
| Extraversion | .022 | -.021 | -.088 | .062 |  |
| Emotionality | -.034 | .054 | .022 | -.054 |  |
| Honesty Humility | -.005 | -.029 | -.040 | -.033 |  |
| NFC | -.025 | .076 | .054 | -.006 |  |
| SDO | .101* | .041 | .094* | .153*** |  |
| RWA | -.119** | .239*** | .235*** | .025 |  |
| Positive attitudes AI | .663*** | -.257*** | -.067 | .642*** |  |
| Negative attitudes AI | -.184*** | .513*** | .512*** | -.186*** |  |

*Note.* In Gender 1 = men, 2 = women.

From the previous table, we can see correlation coefficients between the dimensions of attitudes towards ChatGPT and the rest of the variables in the study.

**Fig. 1C. Participants' education level**

**Fig. 2C. Participants' gender**

## **D. Study 2**

**Table 1D**

*Descriptive statistics of variables for the German sample*

| Variables | *Min* | *Max* | *M* | *SD* |
| --- | --- | --- | --- | --- |
| AI Literacy | 0.00 | 4.00 | 2.79 | 1.00 |
| NFC | 1.00 | 4.11 | 1.96 | 0.87 |
| SDO | 1.00 | 7.00 | 3.29 | 1.25 |
| RWA | 1.40 | 4.80 | 3.46 | 0.63 |
| Openness | 2.25 | 5.00 | 3.71 | 0.70 |
| Conscientiousness | 2.00 | 5.00 | 3.39 | 0.52 |
| Agreeableness | 1.50 | 4.50 | 2.97 | 0.59 |
| Extraversion | 1.75 | 5.00 | 3.63 | 0.63 |
| Emotionality | 1.00 | 4.75 | 2.97 | 0.75 |
| Honesty-Humility | 1.00 | 5.00 | 3.69 | 0.75 |
| Positive attitudes | 1.92 | 5.00 | 3.84 | 0.60 |
| Negative attitudes | 1.00 | 4.88 | 2.69 | 0.72 |
| ChatGPT as a personal assistant | 2.43 | 5.00 | 4.18 | 0.59 |
| ChatGPT as a cognitive impairment risk | 1.00 | 5.00 | 2.67 | 1.00 |
| ChatGPT as a danger humanity | 1.00 | 5.00 | 2.62 | 0.84 |
| ChatGPT as a learning tool | 2.00 | 5.00 | 3.88 | 0.83 |

**Table 2D**

*Correlations between variables in the study—German subsample*

|  | ChatGPT as a personal assistant | ChatGPT as a cognitive impairment risk | ChatGPT as a danger to humanity | ChatGPT as a learning tool |
| --- | --- | --- | --- | --- |
| AI Literacy | .117 | -.148 | -.275^**^ | -.034 |
| Gender | -.057 | .218^*^ | .108 | -.015 |
| Openness | .052 | -.270^**^ | -.250^**^ | .221^*^ |
| Conscientiousness | .198^*^ | -.139 | -.082 | .245^**^ |
| Agreeableness | .011 | .071 | .006 | .091 |
| Extraversion | .169 | .051 | -.020 | .106 |
| Emotionality | -.026 | .053 | .070 | -.130 |
| Honesty Humility | .112 | -.183 | -.158 | .012 |
| NFC | -.177 | .392^**^ | .316^**^ | .041 |
| SDO | -.162 | .121 | .138 | .075 |
| RWA | .091 | .063 | .013 | .029 |
| Positive attitudes AI | .557^**^ | -.098 | -.141 | .567^**^ |
| Negative attitudes AI | -.156 | .467^**^ | .495^**^ | -.259^**^ |

Figure 1D and 2D present sample characteristics of Serbian participants in study 2.

**Fig. 1D. Participants' education level (srb)**

**Fig. 2D. Participants' gender (srb)**

Figure 3D and 4D present sample characteristics of German participants in study 2.

**Fig. 3D. Participants' education level (ger)**

**Fig. 3D. Participants' gender (ger)**

The next table (Table 3D) shows the path analysis on the German sample by gender. Due to the small sample size, this can be considered only as an exploratory analysis. Future studies would need to confirm this on a larger and more representative sample.

**Table 3D**

**Path model by gender on the German sample**

| Criterion | Direction | Predictor | Men | | Women | |
| --- | --- | --- | --- | --- | --- | --- |
|  |  |  | Estimate | *p* | Estimate | *p* |
| Positive attitudes | <--- | Openness | .807 | .007 | -.745 | .041 |
| Positive attitudes | <--- | AI_OPEN | -.612 | .045 | .873 | .022 |
| Negative attitudes | <--- | RWA | .387 | .001 | -.023 | .897 |
| ChatGPT as a cognitive impairment risk | <--- | Negative attitudes | .290 | .011 | .426 | .001 |
| ChatGPT as danger to humanity | <--- | Negative attitudes | .392 | *** | .534 | *** |
| ChatGPT as a learning tool | <--- | Negative attitudes | -.030 | .730 | -.258 | .004 |
| ChatGPT as a personal assistant | <--- | Positive attitudes | .601 | *** | .536 | *** |
| ChatGPT as a learning tool | <--- | Positive attitudes | .632 | *** | .491 | *** |
| ChatGPT as a learning tool | <--- | AI Literacy | -.250 | .006 | -.145 | .211 |
| ChatGPT as danger to humanity | <--- | AI Literacy | -.342 | .001 | -.193 | .124 |
| ChatGPT as danger to humanity | <--- | Extraversion | .058 | .576 | -.212 | .048 |
| ChatGPT as a learning tool | <--- | Openness | .236 | .003 | .162 | .128 |
| ChatGPT as a learning tool | <--- | RWA | .023 | .800 | .443 | *** |
| ChatGPT as a personal assistant | <--- | SDO | -.255 | .006 | -.146 | .228 |
| ChatGPT as a learning tool | <--- | SDO | .095 | .306 | -.345 | .001 |
| ChatGPT as a cognitive impairment risk | <--- | RWA | .352 | .003 | .052 | .683 |
| ChatGPT as a personal assistant | <--- | NFC | -.033 | .693 | .304 | .005 |
| ChatGPT as a learning tool | <--- | Agreeableness | -.079 | .755 | -.447 | .042 |
| ChatGPT as a learning tool | <--- | AI_AGRE | .240 | .342 | .489 | .033 |
| ChatGPT as a cognitive impairment risk | <--- | AI_AGRE | .125 | .687 | .786 | .028 |

The above model (presented in the Table 3D), produced without insignificant paths, showed an adequate fit (Χ² = 223.382, *df* = 186, p = .032. Χ²/*df* = 1.201, GFI = .853, TLI = .951, IFI = .976, CFI = .974, RMSEA .04, SRMR = .08). All fit indices achieved adequate levels except for GFI, being somewhat below the border. However, since most of the indices indicate an adequate model fit, we can conclude that the model is adequate.

Men and women show different predictors for their attitudes towards ChatGPT. Openness is linked to more positive attitudes in men but less in women, while AI openness has the opposite effect—reducing positivity in men but increasing it in women. Negative attitudes are strongly related to RWA in men, but this relationship is absent in women.

Negative attitudes, in turn, are associated with seeing ChatGPT as both a cognitive impairment risk and a danger to humanity, with stronger effects for women. AI literacy is linked to lower perceptions of ChatGPT as a threat, particularly for men. On the contrary, extraversion relates to higher perceptions of ChatGPT as a danger to humanity only in women.

When it comes to viewing ChatGPT as a useful tool, positive attitudes are associated with its acceptance as a personal assistant and a learning tool across both gender groupss. However, women who score higher in RWA are also more likely to see it as a learning tool, while higher SDO positively links to this perception. Additionally, men who are more open tend to see ChatGPT as useful for learning, whereas lower agreeableness in women negatively links to this view.

Need for cognition closure plays a role in how women use ChatGPT, with higher NFC predicting greater likelihood of using it as a personal assistant. Lastly, AI-related attitudes show some gendered effects—higher agreeableness is positively related to the perception of ChatGPT as a cognitive impairment risk, but only for women.

The interaction effects are presented in Figure 4D.

**Fig. 4D. Interaction in the model - German subsample**

Figure 4Da reveals that among the participants with low AI literacy levels, those with high openness exhibit more positive attitudes. The results for individuals with low AI literacy levels are reversed, with the differences between with high and low openness individuals among those with high AI literacy levels being not so extreme.

Figure 4Db reveals that among individuals with low AI literacy, those with low agreeableness tend to view GPT as a learning tool more than the ones with high agreeableness. Among those with high AI literacy differences are negligible.

Figure 4Dc reveals that individuals with low AI literacy and low agreeableness scores tend to view ChatGPT as a cognitive impairment risk more than those with low AI literacy and high agreeableness. Among those with high AI literacy levels the results are reversed, to a smaller extent.

In conclusion, people with low AI literacy rely more on personality traits when forming opinions about ChatGPT. Open-minded individuals in this group have more positive attitudes, while less agreeable ones are more likely to see ChatGPT as a useful learning tool but also a cognitive risk. Among those with high AI literacy, these personality differences matter much less.

## **E. ChatGPT attitudes scales**

**Table 1E**

*The final version of the scale based on the first EFA for two dimensions*

| **Positive aspects** | **Negative aspects** |
| --- | --- |
| 1. Can be very useful if regulated | 2. Will be used by students to write their term papers which will negatively impact education |
| 3. Can be used to stimulate innovation in organizations | 4. Will make some jobs redundant |
| 5. Can be used to stimulate innovation in education | 6. Is very dangerous |
| 7. Can improve efficiency in the workplace | 8. Can be a weapon of mass destruction |
| 9. Can improve efficiency in the education | 10. Can help people to commit crimes which will lead to a higher crime rate |
| 11. Can help with repetitive tasks | 12. It can be misused |
| 13. Can make work easier | 14. You can reach information on how to harm yourself or others more easily |
| 15. It can be helpful in information collection | 16. The human brain will atrophy from the lack of usage |
| 17. It can be helpful in learning about fields other than your own | 18. It threatens humans because it learns, and it can become smarter than us |
| 19. It can help to learn a new language | 20. Cognitive functions in children can develop more slowly |
| 21. It will remove unnecessary steps in some jobs | 22. Critical thinking can become extinct |
| 23. It can be helpful to finish up administrative staff | 24. It will foster laziness |
| 25. It is a very fast encyclopedia |  |
| 26. It helps with some types of jobs like content writing |  |
| 27. It helps to explain stuff step by step |  |
| 28. It is there to help us when we lack the inspiration |  |
| 29. It is there to help us with formal speech |  |
| 30. It responds very fast |  |

**Table 2E**

*Final items based on EFA and CFA for four factors*

| 1. | Can be used to stimulate innovation in organizations. |
| --- | --- |
| 2. | Can be used to stimulate innovation in education. |
| 3. | Can improve efficiency in education. |
| 4. | Can help with repetitive tasks. |
| 5. | Can help people to commit crimes which will lead to a higher crime rate. |
| 6. | Can make work easier. |
| 7. | It can be helpful in information collection. |
| 8. | You can reach information on how to harm yourself or others more easily. |
| 9. | It will remove unnecessary steps in some jobs. |
| 10. | The human brain will atrophy from the lack of usage. |
| 11. | It can be helpful to finish up administrative staff. |
| 12. | It is a very fast encyclopedia. |
| 13. | It helps foster creativity. |
| 14. | It threatens humans because it learns, and it can become smarter than us. |
| 15. | Cognitive functions in children can develop more slowly. |
| 16. | Critical thinking can become extinct. |
| 17. | It will foster laziness. |

Scoring key:

ChatGPT as a personal assistant: 1, 4, 6, 7, 9, 11, 12; ChatGPT as a cognitive impairment risk: 10, 15, 16, 17; ChatGPT as a danger to humanity: 5, 8, 14; and ChatGPT as a learning tool: 2, 3, 13.
